# Whole-genome sequence of the bovine blood fluke *Schistosoma bovis* supports interspecific hybridization with *S*. *haematobium*

**DOI:** 10.1371/journal.ppat.1007513

**Published:** 2019-01-23

**Authors:** Harald Oey, Martha Zakrzewski, Kerstin Gravermann, Neil D. Young, Pasi K. Korhonen, Geoffrey N. Gobert, Sujeevi Nawaratna, Shihab Hasan, David M. Martínez, Hong You, Martin Lavin, Malcolm K. Jones, Mark A. Ragan, Jens Stoye, Ana Oleaga, Aidan M. Emery, Bonnie L. Webster, David Rollinson, Robin B. Gasser, Donald P. McManus, Lutz Krause

**Affiliations:** 1 The University of Queensland Diamantina Institute, The University of Queensland, Brisbane, QLD, Australia; 2 Genetics & Computational Biology Department, QIMR Berghofer Medical Research Institute, Brisbane, QLD, Australia; 3 Faculty of Technology and Center for Biotechnology (CeBiTec), Bielefeld University, Bielefeld, Germany; 4 Faculty of Veterinary and Agricultural Sciences, The University of Melbourne, Parkville, VIC, Australia; 5 Immunology Department, QIMR Berghofer Medical Research Institute, Brisbane, QLD, Australia; 6 School of Biological Sciences, Queen’s University Belfast, Belfast, Northern Ireland, United Kingdom; 7 UQ Centre for Clinical Research, The University of Queensland, Brisbane, QLD, Australia; 8 School of Veterinary Science, University of Queensland, Gatton, QLD, Australia; 9 Institute for Molecular Bioscience, The University of Queensland, Brisbane, QLD, Australia; 10 Institute of Natural Resources and Agrobiology (IRNASA, CSIC), Cordel de Merinas, Salamanca, Spain; 11 Natural History Museum, Life Sciences Department, Parasites and Vectors Division, Cromwell Road, London, United Kingdom; University of Texas Rio Grande Valley, UNITED STATES

## Abstract

Mesenteric infection by the parasitic blood fluke *Schistosoma bovis* is a common veterinary problem in Africa and the Middle East and occasionally in the Mediterranean Region. The species also has the ability to form interspecific hybrids with the human parasite *S*. *haematobium* with natural hybridisation observed in West Africa, presenting possible zoonotic transmission. Additionally, this exchange of alleles between species may dramatically influence disease dynamics and parasite evolution. We have generated a 374 Mb assembly of the *S*. *bovis* genome using Illumina and PacBio-based technologies. Despite infecting different hosts and organs, the genome sequences of *S*. *bovis* and *S*. *haematobium* appeared strikingly similar with 97% sequence identity. The two species share 98% of protein-coding genes, with an average sequence identity of 97.3% at the amino acid level. Genome comparison identified large continuous parts of the genome (up to several 100 kb) showing almost 100% sequence identity between *S*. *bovis* and *S*. *haematobium*. It is unlikely that this is a result of genome conservation and provides further evidence of natural interspecific hybridization between *S*. *bovis* and *S*. *haematobium*. Our results suggest that foreign DNA obtained by interspecific hybridization was maintained in the population through multiple meiosis cycles and that hybrids were sexually reproductive, producing viable offspring. The *S*. *bovis* genome assembly forms a highly valuable resource for studying schistosome evolution and exploring genetic regions that are associated with species-specific phenotypic traits.

## Author summary

In this article we detail the assembly and functional annotation of the *Schistosoma bovis* genome. *S*. *bovis* is a parasitic flatworm that primarily infects bovines, with important economic consequences in affected countries. However, it is also a close relative of the human carcinogenic parasite *Schistosoma haematobium* which is a serious health issue in many endemic countries in Sub-Saharan Africa. The close relationship and overlapping geographical distribution of *S*. *bovis* and *S*. *haematobium* allows these to hybridise in the wild increasing their genetic diversity and presenting the risk of zoonotic transmission, i.e. the transmission from animals to humans. The hybridization between human and ruminant schistosomes is of particular interest as interspecific hybridization may have dramatic impacts on transmission rates, disease dynamics, control interventions and parasite evolution. By whole-genome sequencing and comparative genomics we present evidence that fertile hybrids are indeed present in the wild, presenting the potential risk of transmission from animal reservoirs to humans.

## Introduction

Schistosomiasis is a neglected tropical disease caused by parasitic flatworms of the genus *Schistosoma*, infecting both humans and animals as definitive hosts [1]. *Schistosoma bovis* causes intestinal schistosomiasis in cattle, sheep and goats and is one of the most significant veterinary problems in African countries [2]. In Sub-Saharan Africa high prevalence rates of chronic schistosome infections in some endemic areas cause significant losses attributable to reduced growth and productivity, increased susceptibility to other infectious agents, and death [3]. The parasite is transmitted to ruminants from freshwater snails. After penetrating the skin, larvae undergo a complex development into dioecious adult egg-laying worm pairs. Adult flukes are found in the portal, mesenteric, and intestinal submucosal and subserosal veins from within which the females release eggs that become embedded in the intestinal wall and other tissues, or are excreted in stool, contributing to disease transmission.

*S*. *bovis* has recently come into the spotlight as a possible emerging health threat following the molecular identification of *Schistosoma haematobium-bovis* hybrids from children in Senegal [4] and during a recent schistosomiasis outbreak in Corsica [5]. The human-infecting schistosome *S*. *haematobium* is a major source of urogenital disease [6], causes squamous cell carcinoma in the bladder [5, 7], and is a predisposing factor for HIV/AIDS [8]. The hybridization between human and ruminant schistosomes is of particular interest, as interspecific hybridization may have dramatic impacts on disease dynamics, transmission rates and parasite evolution. It has been shown that laboratory hybrids acquire enhanced characteristics, including increased infectivity, growth rates, maturation and egg production [9]. These findings underscore the need for improving our knowledge of veterinary schistosome species, not only for improved disease control in animals, but also to prevent potential transmission of hybrid species to humans from animal reservoirs. Here, we report a high-quality assembly of the genome of *S*. *bovis*, which provides a valuable resource for studying its biology and enabling comparative schistosome genomic research.

## Results and discussion

### Genome assembly

**Fig 1:**
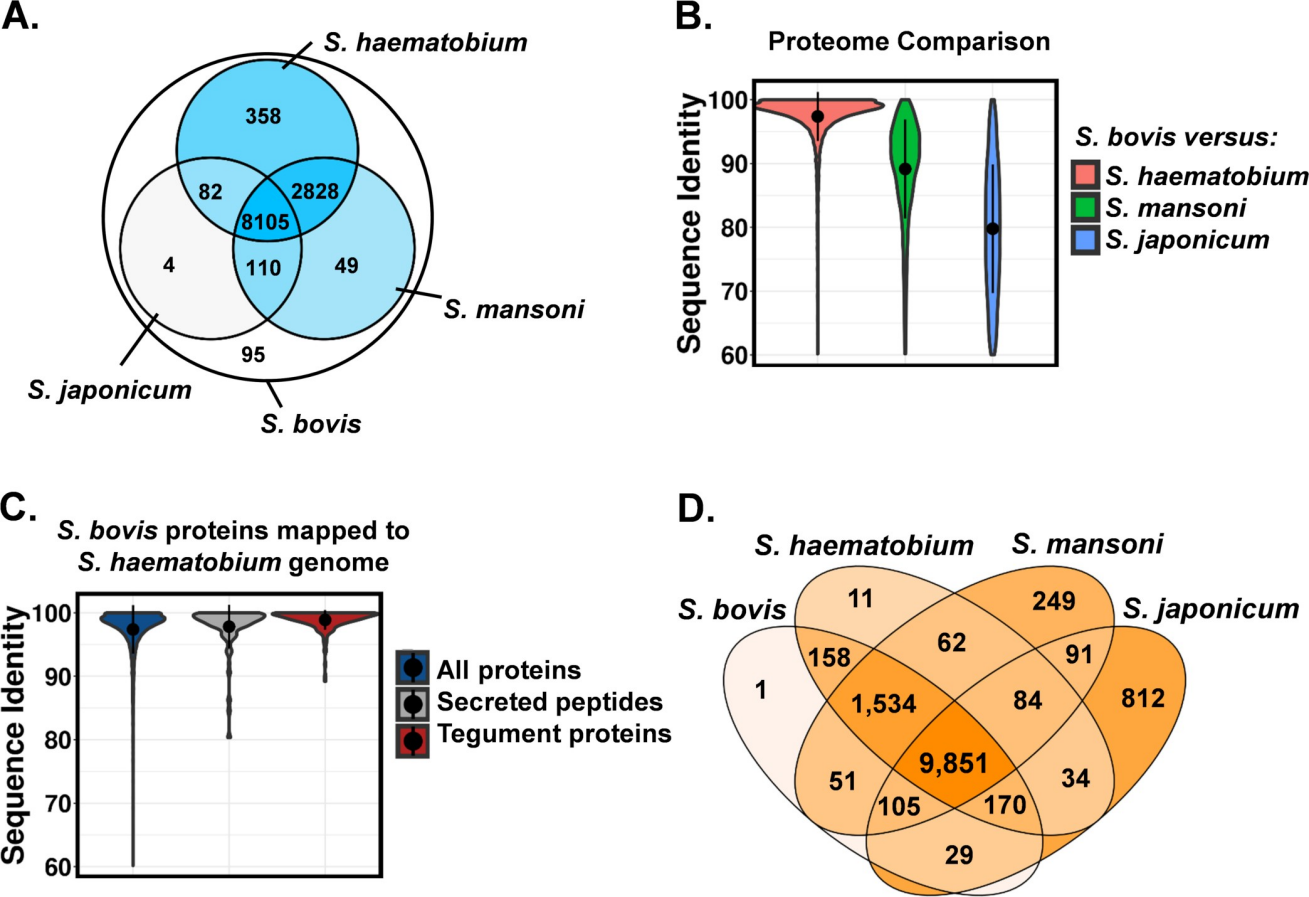
Comparison of the *S*. *bovis* proteome with other schistosome species. A) *S*. *bovis* centred Venn diagram. The outer ring represents the 11,631 proteins predicted from the *S*. *bovis* genome, while the inner 3-way Venn diagram shows the subset of predicted *S*. *bovis* proteins that were present in the genomes of *S*. *haematobium* (Egypt isolate), *S*. *mansoni and S*. *japonicum*, respectively, based on protein sequence homology. The colours of the inner 3-way Venn diagram correspond to the number of proteins in each intersection from low (light grey) to high (dark blue). A *Schistosoma* core-proteome of 8,105 proteins was identified as those proteins that had sequence homology matches across all four species. 95 predicted proteins were unique for *S*. *bovis*. B) Sequence identity of *S*. *bovis* proteins and orthologous proteins from other schistosome species. Proteins of *S*. *bovis* and *S*. *haematobium* were significantly more conserved (p<10^−15^, two-tailed t-test) than the proteomes of *S*. *bovis-S*.*-mansoni* and *S*. *bovis-S*. *japonicum*. C) Sequence conservation of *S*. *bovis* tegument proteins and secreted peptides. *S*. *bovis* proteins were mapped to the *S*. *haematobium* genome sequence using Exonerate. Tegument proteins and secreted peptides showed an average sequence identity of 98.9% and 97.8%, respectively. All tegument proteins and secreted peptides included in this analysis were shared by both species. D) Analysis of the *Schistosoma* pan-genome. Proteomes of four *Schistosoma* species were concatenated, clustered into orthologous groups and mapped to each *Schistosoma* genome using Exonerate. Colours depict the number of proteins in each intersection from low (light orange) to high (dark orange). A pan-genome of 13,297 orthologous groups and a *Schistosoma* core-genome of 9,851 proteins were identified.

*Schistosoma bovis* eggs were obtained from the liver of a routinely slaughtered infected cow in 1997 in Iringa, Tanzania. The eggs were put into fresh water and hatched into miracidia that were used to infect laboratory-maintained *Bulinus wrighti* snails and the resulting cercariae were used to establish the *S*. *bovis* isolate in laboratory passage at the Natural History Museum, London using mice as the definitive host. Adult worm pairs were perfused from infected mice and frozen in liquid nitrogen as a future genetic resource [10]. A mated adult schistosome worm pair (single male and female) from the 5th laboratory passage of this isolate was used for genome sequencing, yielding 1.9 μg of genomic DNA. Short-insert (200 bp and 500 bp) and mate-pair (800 bp, 2 kb and 5 kb) genomic DNA libraries were paired-end sequenced on the Illumina HiSeq platform, yielding 56.1 Gb of data (**Table A and Table B in S1 Text**). Additionally, 2.3 Gb of long-read data were generated on the PacBio platform, providing reads with an average length of 3.2 kb (**Table A and Fig A in S1 Text**). The distributions of 17-mer coverage in the short-read data presented with single peaks (**Fig B in S1 Text**) suggesting low heterozygosity as high heterozygosity would produce bimodal or multimodal distributions [11]. The genome size was estimated at 388–392 Mb by the program GenomeScope [12]. GenomeScope fits a mixture model of negative binomial distributions to the k-mer profile, in order to measure the relative abundances of heterozygous and homozygous k-mers. K-mer frequencies of 21 and 23 were used as input, respectively. Sequence data were assembled into a 374 Mb genome sequence (4,780 scaffolds with N50 of 203kb) (**Table 1)**. We identified a large number of repetitive regions, accounting for 35.8% of the *S*. *bovis* genome. In accordance with other *Schistosoma* species [13–16], retrotransposons of the long interspersed nuclear element (LINE) subtype were the major class of repetitive elements comprising 17% of the genome. Additionally, we identified short interspersed elements (SINEs) (4% of genome) and long terminal repeat (LTR) retrotransposons (4%). Another 11% of the genome consisted of unclassified repeats (**Table C in S1 Text)**. Genes were predicted using a combination of intrinsic and similarity-based methods and identified 11,631 protein encoding sequences, a number comparable to other schistosomes (range 11,774 to 12,657) (**Table 1**). The majority of genes predicted in the *S*. *bovis* genome have homologs in the genomes of *S*. *haematobium* (98.3%), *S*. *mansoni* (96.0%) and *S*. *japonicum* (71.9%) (**Fig 1A**).

**Table 1:** Characteristics of *S*. *bovis*, *S*. *haematobium* (Egypt isolate) [15], *S*. *mansoni* [13] and *S*. *japonicum* [16] genome assemblies.

	*S*. *bovis*	*S*. *haematobium*	*S*. *mansoni*	*S*. *japonicum*
**Estimated genome size (Mb)**	388–392^c^	385	381	403
**Number of protein coding genes**	11,631	11,140	11,774	12,657
**GC content (%)**	34.4	32.1	34.7	33.5
**Repeat rate (%)**	35.83	34.44	39.66	36.85
***Contig statistics***				
**Total base pairs (Mb)**	361.0	352.7	362.5	369.0
**Number of contigs**	20,949	59,154	9,520	95,338
**N50 length (kb)**	34.9	22.4	76.7	6.1
**Length largest contig (kb)**	423.3	180.9	459.9	92.5
**Mean contig size (kb)**	17.2	6.0	38.1	3.9
**Median contig size (kb)**	9.6	0.9	20.9	2.5
***Scaffold statistics***				
**Number of scaffolds**	4,780	29,834	885	25,048
**Assembly size (Mb)**^a^	373.7	375.9	364.5	397.7
**Assembly total base pairs (Mb)**^b^	361.0	352.7	362.5	369.0
**N50 length (kb)**	202.9	317.5	32,115.3	173.6
**Length largest scaffold (Mb)**	1.1	1.8	65.5	1.7
**Number > 1kb (% of assembly)**	4,780 (100%)	7,462 (97.6%)	884 (99.9%)	25,029 (100%)
**Number > 10kb (% of assembly)**	4,367 (99.3%)	2,384 (94.5%)	446 (99.4%)	4,663 (87.0%)
**Number > 100kb (% of assembly)**	1,045 (70.9%)	958 (79.8%)	158 (97.0%)	795 (59.3%
**Mean scaffold size (kb)**	78.2	12.6	411.9	15.9
**Median scaffold size (kb)**	27.7	0.43	10.4	2.1

^a^Combined length of all scaffolds in Mb;

^b^Combined length of all scaffolds without gaps (N’s) in Mb;

^c^Estimated by GenomeScope

### Genome comparison

The genome sequence of *S*. *bovis* is similar to that of the human-infecting schistosomes *S*. *haematobium* (Egypt isolate), *S*. *mansoni* and *S*. *japonicum*, in terms of GC-content (34.4%), repeat-derived DNA (35.8%), total number of genes (11,631), average number of exons per gene (4.8), average exon length (256 bp), average intron length (1.96kb) and functional GO categories (**Table 1 and Table D and Fig C in S1 Text**). The genomes of *S*. *bovis* and *S*. *haematobium* are highly similar, with ~97% sequence similarity across the aligned scaffolds, whereas *S*. *mansoni* and *S*. *japonicum* are more-distantly related to *S*. *bovis* (85% and 70% similarity, respectively), consistent with the present knowledge of the evolutionary relationships of schistosomes [17]. A genome-wide comparison of *S*. *bovis* and *S*. *haematobium* identified 6.9 million single nucleotide substitutions and 320,000 short indels. An analysis of paired-end sequence data further revealed high synteny, identifying only 55 insertions, 9 inversions, 11 intra-chromosomal translocations and 43 inter-chromosomal translocations. Similarly, *S*. *bovis* and *S*. *haematobium* are strikingly similar on the proteome level, with 98.3% of predicted *S*. *bovis* proteins having orthologues in *S*. *haematobium* (**Fig 1A**) and with 96.7% of *S*. *haematobium* proteins also present in *S*. *bovis*. Proteins are highly conserved between the two species, with an average identity of 97.3% on the amino acid level (**Fig 1B**). Unexpectedly, even tegument proteins and secreted peptides are highly conserved (>97.8% average sequence identity), despite their critical role in parasite-host interactions and immune evasion or modulation (**Fig 1C**). Genome comparison of the four schistosome species (*S*. *haematobium*, *S*. *bovis*, *S*. *mansoni and S*. *japonicum*) identified a *Schistosoma* pan-genome of 13,297 inferred orthologous groups and a core-genome of 9,851 groups (**Fig 1D**). *S*. *bovis* and *S*. *haematobium* share 96.9% of orthologous groups. As expected, *S*. *japonicum* was genetically the most distinct species, showing a high number of species-specific proteins (812) and missing 1,534 groups that were relatively conserved across the other three schistosome species. In light of the limited genomic and proteomic differences between *S*. *bovis* and *S*. *haematobium*, it is likely that speciation, host-specificity, organ-specificity and differences in morphology are driven by minor genetic modifications of existing genes and regulatory motifs as well as post-translational modifications of proteins (e.g. glycosylation).

**Fig 2:**
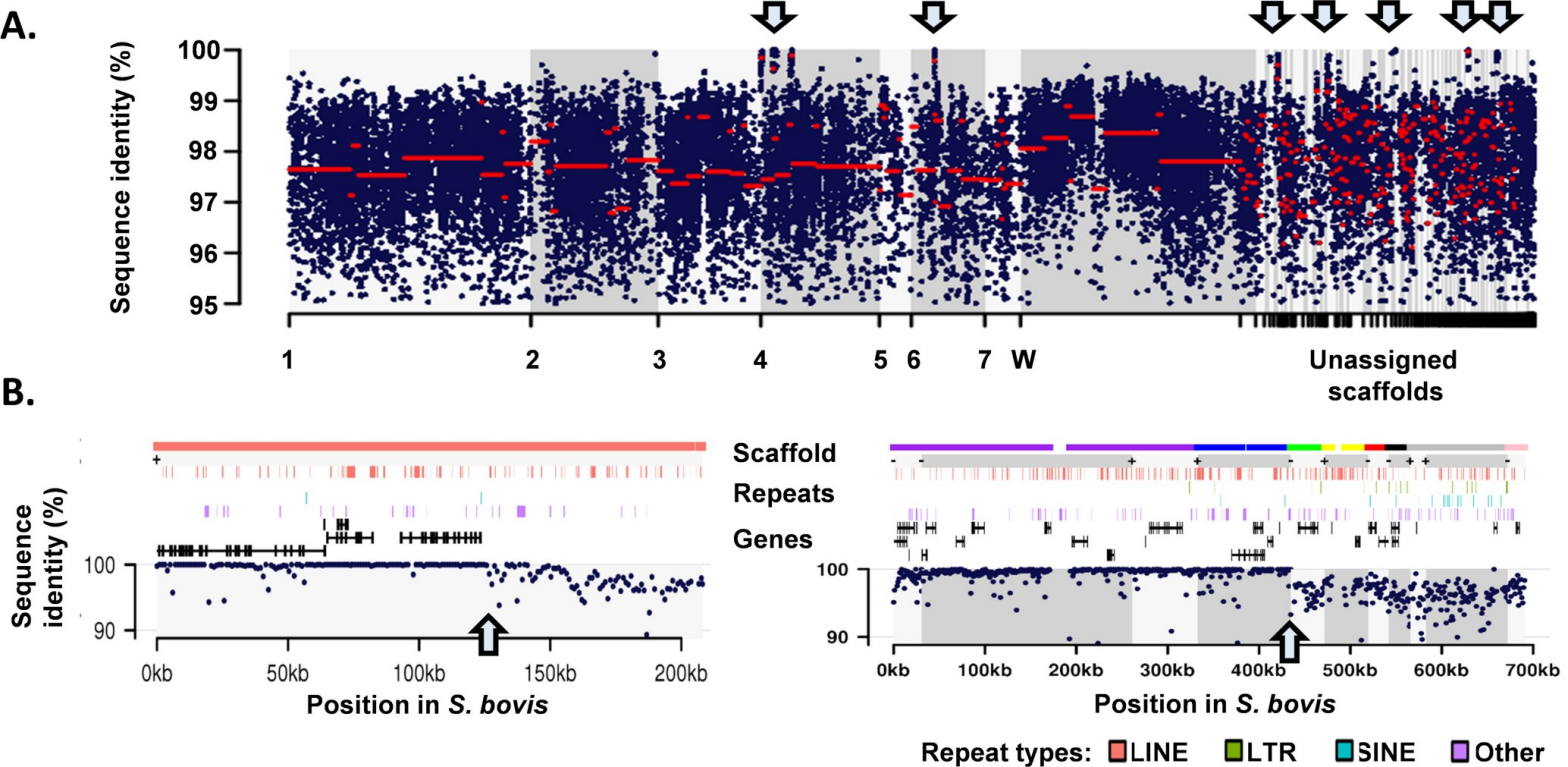
Whole-genome comparison of *S*. *bovis* and *S*. *haematobium*. A) Whole-genome comparison of *S*. *bovis* and *S*. *haematobium* (Egypt isolate) reveals remarkably high sequence similarity and provides evidence of interspecific hybridization. Pairwise alignments were carried out using mafft and the sequence identity calculated for 1 kb windows across the resulting alignments. Chromosome numbers were assigned by mapping *S*. *bovis* scaffolds to the *S*. *mansoni* genome assembly (autosomal chromosomes 1–7 and sex chromosome W). Regions with very high sequence similarity (sequence identity >99%) provide evidence of recent interspecific hybridization and are marked by blue arrows. Red lines depict segments with similar similarity levels, which were found by fitting a piecewise constant curve using a least-squares cost function. Grey shaded regions indicate mauve alignment blocks. The top coloured bar represents the *S*. *bovis* scaffold of each 1kb window and gaps represent regions of the *S*. *bovis* genome that were not aligned to the *S*. *haematobium* genome using mafft. B) Two remarkably similar regions on chromosome 4. Arrows mark breakpoints between highly similar and less-similar genomic regions. Top coloured bar represents *S*. *bovis* scaffolds and orientation (+/-). Repetitive elements (LINEs, LTRs, SINEs and others) are shown as coloured bars. The regions span protein-coding genes, intergenic regions and repetitive elements. High sequence similarity of repeats is indicative of recent *S*. *bovis-haematobium* hybridization, as the lower selective pressure acting on repetitive elements would otherwise lead to a rapid accumulation of mutations.

Remarkably, the genomes of the *S*. *bovis* isolate and the Egypt *S*. *haematobium* isolate showed distinct regions with >99% sequence identity over long stretches of DNA with some spanning several hundred kb (**Fig 2A and 2B**). The corresponding regions in *S*. *mansoni* and *S*. *japonicum* did not show a comparable effect, and we did not identify other regions in these genomes with such high sequence similarity (**Fig D panel A and panel B in S1 Text**), suggesting that this is a unique feature of the *S*. *bovis* and *S*. *haematobium* isolates investigated herein. Normally, sequence conservation between species is confined to discreet regions under selective pressure, such as genes and regulatory elements [18]. Additionally, short and highly conserved non-coding elements, known as ultraconserved elements, many of which have no known function, have also been identified in whole-genome alignments of other species [19, 20]. However, the length of the very similar regions between *S*. *bovis* and *S*. *haematobium* and the lack of conservation when compared to more distantly related schistosome species are not consistent with such elements. Instead, the observed regions span large stretches of contiguous sequence and are not linked to distinct genetic elements. The high degree of similarity also extends through repetitive elements that are not typically under purifying selection [21]. *S*. *bovis* and *S*. *haematobium* have been reported to hybridize in the wild and the high genetic similarity is evidence of recent *bovis-haematobium* hybridization, resulting in interspecific genetic exchanges. The results indicate that the incorporated alleles have been passed through multiple meiosis cycles and that therefore *S*. *bovis-haematobium* hybrids are sexually reproductive, giving rise to viable and fertile offspring.

The precise timing of the hybridization event is not clear. However, as only short sub-chromosomal regions were identified, the event must have occurred several generations ago, as the F1 generation would have had 50% of its DNA derived from either parent (i.e. the entire genome would be heterozygous). Subsequent back-crosses to non-hybrid individuals then resulted in the gradual loss of the hybrid chromosomes. However, while whole chromosomes are lost at a constant rate through back-crossing in the wild, sub-chromosomal regions are exchanged through meiotic recombination and can persist for longer and may even become fixed, particularly in restricted or inbred populations.

The nature of the highly similar genomic regions was further characterized by mapping sequencing reads from our *S*. *bovis* isolate and the previously published Egypt *S*. *haematobium* isolate [15] to both the respective *Schistosoma* genome assemblies. To infer the potential direction of the hybridization event we also included recently released exome-sequence datasets of eight *S*. *haematobium* strains isolated from the Zanzibar archipelago [22]. Sequencing reads from all 10 datasets were aligned to the *S*. *bovis* and *S*. *haematobium* genome assemblies and variants were called using the GATK UnifiedGenotyper [23] on all datasets simultaneously such that the allele status would be reported for all samples at all positions with sufficient coverage. As expected for a region shared through hybridisation, the Egypt *S*. *haematobium* isolate showed lower allele frequencies (i.e. most variants have variant allele frequencies of 0) for variants located within the highly similar genomic regions than for variants located outside of these regions (i.e. variants flanking the regions have variant allele frequencies of either 0 or 1), when mapped to *S*. *bovis* (**Fig E panel A and panel B and Fig F panel A and panel B in S1 Text**). Interestingly, the eight Zanzibar *S*. *haematobium* isolates did not show this characteristic pattern. Based on these results we propose that the very similar genomic regions represent segments of *S*. *bovis* DNA that have been acquired by the Egypt *S*. *haematobium* isolate via interspecific hybridization. Variants called when mapping the same reads to *S*. *haematobium* support this hypothesis with the 8 Zanzibar *S*. *haematobium* isolates showing higher variant allele frequencies for variants located within the highly similar genomic regions than for variants located outside of these regions (**Fig E panel A and panel B and Fig F panel A and panel B in S1 Text**). While, together, these results suggest that the genome of the Egypt *S*. *haematobium* isolate harboured some foreign *S*. *bovis* DNA, the limited resolution of the studied exome-sequence datasets and the low number of variants located within the highly similar regions does not allow us to make any definitive conclusions regarding the direction of the hybridization. Furthermore, sequencing of additional *S*. *bovis* isolates would be required to determine if the regions in the *S*. *bovis* genome presented herein are also representative of other *S*. *bovis* isolates.

Interestingly, we observed a high variability in the level of sequence similarity along the sex-chromosome (W chromosome) (**Fig 2A**), a pattern that was not observed for the remaining (autosomal) chromosomes. This profile in sequence similarity could be explained by the fact that some parts of the W chromosome show reduced rates of recombination [24].

Despite the remarkably high similarity at both the genomic and proteomic levels between the two species and their ability to hybridize, *S*. *bovis* and *S*. *haematobium* are sister but distinct species, based on characteristics including morphology, host specificity and life-cycle characteristics [25]. These results demonstrate the challenges in defining different species or species complexes in schistosomes.

*S*. *haematobium* is recognized as a definite cause of bladder cancer and continuous deposition of *S*. *haematobium* eggs in the bladder can lead to squamous cell carcinoma [1]. Mechanical damage and the release of toxic parasite excretory/secretory (ES) molecules have been implicated in the mechanisms that induce cancer in the epithelial cells of the urinary bladder tract [2]. Secretion of glycoprotein Omega-1 from *Schistosoma* eggs is thought to trigger a Th2-type immune response leading to a cancerous environment [3, 4]. Also estradiol 17beta-dehydrogenase has been proposed to be associated with tumorigenesis in *S*. *haematobium*, potentially via oestrogen receptor-mediated cell proliferation [5]. We found highly conserved homologues of both *S*. *haematobium* proteins in *S*. *bovis*, with sequence identities of 92% and 98%, respectively (**Table E and Fig G in S1 Text**). It is possible that minor changes in protein sequences as well as the infected organ, location of egg deposition and the specific spatial (tissue) and temporal expression of proteins contribute to carcinogenesis.

Schistosomes have been targeted for elimination by the World Health Organization, and vaccines that induce long-term protective immunity represent a logical component for the future control of schistosomiasis. Only three schistosome vaccine antigens have entered human clinical trials (Sm14, Sm-TSP-2 and Sh28GST) [26, 27] and another molecule (Smp80) will likely advance to clinical development [27]. We identified highly conserved *S*. *bovis* orthologues of Sm14, Sh28GST and Sm-p80 (97.2–99.0% sequence identity) and a moderately conserved orthologue of Sm-TSP-2 (34.1%) (**Supplementary results and Table F in S1 Text**). These results indicate that existing *Schistosoma* vaccine targets represent promising vaccine target candidates against *S*. *bovis*.

### Phylogenetic analysis and divergence time

**Fig 3:**
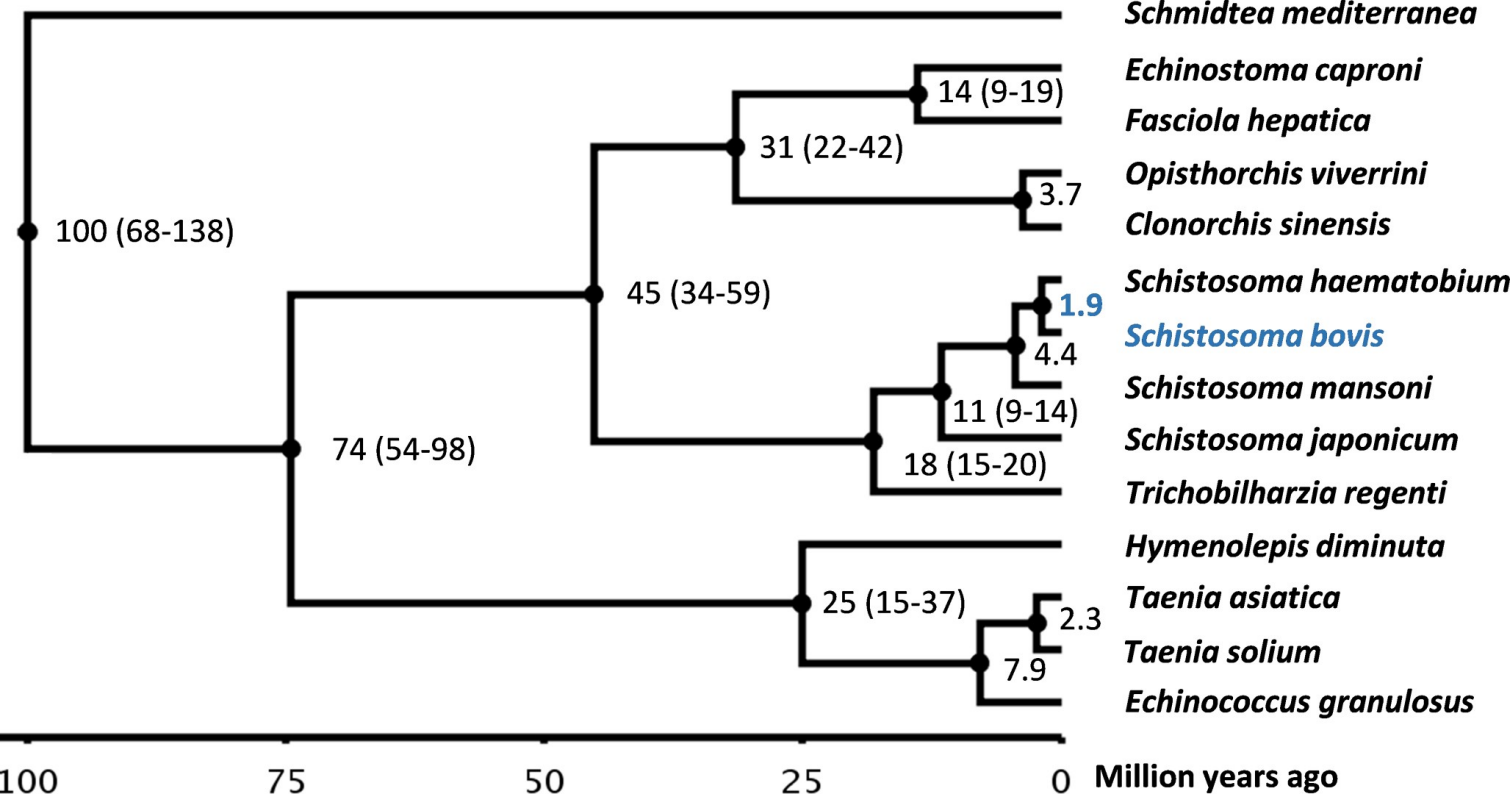
Phylogenetic tree and estimated divergence times. A maximum-likelihood phylogenetic tree was inferred from concatenated protein sequences corresponding to 52 shared single-copy genes. Species divergence was estimated using a Bayesian relaxed molecular clock model. Divergence time is given in million years; 95% confidence intervals are shown in brackets. The divergence time between *S*. *bovis* and *S*. *haematobium* was estimated to be 1.85 million years (95% confidence interval 1.21–2.63 mya). The model was calibrated using previously published divergence times and ages of fossil records of intermediate snail hosts.

We reconstructed a maximum-likelihood phylogenetic tree from concatenated sequences of 52 shared single-copy genes (**Fig H in S1 Text**) and estimated species divergence using a Bayesian relaxed molecular clock model (**Fig 3**). The model was calibrated using previously published divergence times and ages of fossil records of intermediate snail hosts. It has previously been estimated that schistosomes originated in the Miocene around 15–20 million years ago (mya) [28, 29]. Based on fossil records of its intermediate host *Biomphalaria*, it has further been estimated that *S*. *mansoni* likely did not occur before 2–5 mya ago [30]. Evidence for trematode infestations have been reported from the Eocene and preserved trematode eggs have been found in dinosaur coprolites from the Early Cretaceous, but fossil records indicate that trematodes may have existed more than 400 mya [31, 32]. The trematode split was therefore fixed at >56 mya. Using the calibrated model the divergence between *S*. *bovis* and *S*. *haematobium* was estimated to have occurred 1.85 mya with a 95% confidence interval of 1.21–2.63 mya (**Fig 3**).

### Mitochondrial genome

The *S*. *bovis* mitochondrial genome sequence was *de-novo* assembled from long PacBio reads, yielding a 20 kb contig **(Fig I in S1 Text)** with >100-times sequencing coverage across the non-repetitive part and close sequence similarity to the 15 kb mitochondrial genome previously published for *S*. *haematobium genome* (accession DQ157222), and almost identical to a partial *S*. *bovis* mitochondrial genome sequence available in GenBank (accession HM594942). As expected, the mitochondrial genomes of the two species showed the same order of genes and the only detectable differences were indels in intergenic regions and single nucleotide substitutions. The mitochondrial genome contains a ~4 kb region of repetitive sequence that has not been previously assembled for *S*. *bovis* or *S*. *haematobium* [33]. The repetitive region is comprised of two repeat units, 520 bp and 460 bp in length, respectively, arranged in an alternating pattern **(Fig I in S1 Text)**. The shorter of the two units contains a sequence predicted to code for a tRNA for the amino acid serine, though it is not known whether these tRNAs are transcriptionally active or serve some other purpose. No sequence similarity was evident between the *S*. *bovis* repetitive region and the repetitive regions of the mitochondrial genomes of other schistosomes for which the repetitive region has been sequenced, such as *S*. *mansoni* [13] and *S*. *spindale* [33] (**S2 Data**), consistent with a high degree of sequence variability shown by others for this region [33]. Long non-coding regions are also found in the mitochondrial genomes of other trematodes, though often these have only been partially sequenced due to the difficulty of sequencing long repetitive regions [34, 35]. The assembled 20 kb *S*. *bovis* mitochondrial contig was not fully circularized with both ends terminating in the repetitive region. Only a single 8 kb PacBio read bridged across the repetitive region harbouring 4 kb of repetitive sequence (**Fig J in S1 Text**). However, some non-spanning PacBio reads contained repetitive sequences exceeding this length (up to 6 kb), indicating that the repetitive region varies in length between or within *S*. *bovis* isolates. The variability was also supported by the 5 kb mate-pair library with only a small number of pairs spanning across the region but with inconsistent spacing of the mates (**Fig K in S1 Text**).

### Conclusions

Our genome assembly presents a valuable resource for studying schistosome evolution and hybridization events. It will be fascinating to further this analysis by examining isolates in areas of West Africa where recent hybridization between *S*. *bovis* and *S*. *haematobium* has been discovered. The genome shows great promise for improving our understanding of veterinary schistosomes, for developing novel disease interventions and sensitive diagnostic tests for disease control. Our results suggest that *S*. *bovis-haematobium* hybrids are sexually reproductive, producing viable offspring and that exchanged alleles are maintained in the population through multiple meiosis cycles. However, despite their ability to hybridize and their remarkably high level of similarity on genomic and proteomic levels, *S*. *bovis* and *S*. *haematobium* are distinct species based on a number of characteristics. Further genome analysis might provide insights into host specificity, morphological differentiation and species specific life-cycle characteristics.

## Methods

### Data

The *S*. *haematobium* genome sequence SchHae_1.0 version GCA_000699445.1 was downloaded from NCBI. The *S*. *japonicum* genome sequence was obtained from SchistoDB version 28. The *S*. *mansoni* genome sequence ASM23792v2.31 was downloaded from ensemblgenomes.org release 31. Raw sequence reads used for *S*. *haematobium* and *S*. *bovis* SNP analyses were obtained from the NCBI Short Read Archive under the accessions SRR433860, SRR6881116, SRR6881117, SRR6881118, SRR6881119, SRR6881120, SRR6881121, SRR6881122, SRR6881123.

### Sample preparation and sequencing

*S*. *bovis* was isolated from a cow in 1997 in Iringa Tanzania and passaged in mice at the Natural History Museum, London. Adult worms were collected at the 5th passage on 26/01/2000 and stored in liquid Nitrogen. The *S*. *bovis* genome was sequenced from a mated pair of adult worms yielding 1.9 ug of genomic DNA. All of the adult parasites were historically archived specimens whose production required no further use of laboratory animal hosts. All were originally passaged at the Natural History Museum, London, in accordance with the UK Animals (Scientific Procedures) Act 1986 under a series of project licences, the most recent of which were PPL 70/4687 (1998–2003), PPL 70/5935 (2003–2008) and PPL 70/6845 (2008–2012). In addition to Home Office approval, the project licences and activities carried out under them were approved by the Natural History Museum ASPA Ethical Review Process Committee. All regulation and inspection was carried out in full compliance with the ASPA legislation of the time. The genomic DNA was sequencing on the Illumina platform, providing 56.1 Gb of sequence information (**Table A and Table B in S1 Text**), and by PacBio sequencing yielding 2.2 Gb of data (**Table A in S1 Text**).

### Genome size estimation

Frequencies of k-mers of lengths 21 and 23bp were calculated for the combined 200bp and 500bp short read libraries using the program Jellyfish, v.2.2.6, with the options count -C -m <k-mer size> -s 1000000000 [36]. The resulting k-mer frequencies were then used as input for the program GenomeScope [12] to estimate the genome size using default parameters.

### Assembly

Illumina data were processed using sickle [37] and ErrorCorrection from the SOAPdenovo2 package, v.r240 [38]. Reads were assembled into contigs with SOAPdenovo2 using a sequential approach, from short to long insert size libraries. Scaffolding was performed by SOAPdenovo2 requiring at least three pairs to connect contigs to a scaffold, and gaps were closed with SOAPdenovo GapCloser, v.1.12-r6. Various options and k-mer lengths ranging between 23 and 55 were run during the assembly process. An assembly with a k-mer size of 29 and option–M 2 was chosen on the basis of high N50 contig length and high coverage of conserved eukaryote genes as assessed by the CEGMA pipeline [39].

PacBio sequence data was corrected using Illumina reads and were assembled by MIRA, v.4.0.1 (with PacBio HQ settings, 3 reads) [40]. The SOAPdenovo assembly of the Illumina data and the MIRA assembly of the PacBio data were combined by gamngs, v.1.1, with the option block-size: 10 [41].

The mitochondrial genome of *S*. *bovis* was *de-novo* assembled from PacBio reads by Canu v.1.3 [42] with the option genomeSize set to the estimated size of the *S*. *bovis* genome. The accuracy of the resulting contig was confirmed by mapping short insert paired-end sequences directly onto the contig with the program Bowtie2, v.2.2.9 [43], and the mapped reads visually inspected. Three discrepancies were identified and manually changed to match the Illumina reads. The short reads could not be accurately mapped to the repetitive region due to the high sequence similarity of the repeats units.

### Repetitive elements

Repeats were characterized *de-novo* for each of the four *Schistosoma* genomes by RepeatModeller, v.1.0.8 [44] with the option -engine ncbi, and the resulting consensus sequences were used to identify repetitive regions using the RepeatMasker program, v.4.0, with the option -no_is [45].

### Gene identification and functional annotation

The Maker v.2.31.8 gene annotation pipeline [46] was employed for gene identification using Augustus, v.3.0.3 [47], with the gene prediction model “schistosoma2” included with the program, and GeneMark [48], v.4.33, trained on the *S*. *bovis* assembly using the gmes_petap.pl–ES function, for *ab-initio* predictions. *S*. *haematobium* ESTs were provided using the ‘*ESTs from an alternative organism*’ option. Additionally, annotated proteins from *S*. *haematobium*, *S*. *mansoni*, *Caenorhabditis elegans*, *Echinococcus granulosus*, *Taenia solium* and Swiss-Prot [49] were provided to Maker for similarity-based searches. Maker was set to allow prediction of single-exon genes and otherwise default parameters.

Protein sequences were functionally annotated against the KEGG family_eukaryotes database using the BlastKOALA program with otherwise default parameters [50]. Additionally, InterProScan v.5.25–64.0 with the options—iprlookup—goterms—pathways [51] was used to identify functional domains, transmembrane proteins and signal peptides. GO annotations were obtained from InterProScan and analyzed using WEGO [52].

Experimentally validated tegument protein sequences (n = 414) and secreted peptides (n = 375) were extracted from the published literature and the NCBI non-redundant proteins database. PISCES [53] was applied to remove proteins with sequence identity over 20% to reduce biases towards overrepresented proteins. A total of 249 surface proteins and 205 secreted proteins was retained (sequences of proteins included are available from: http://cgenome.net/schisto/). Published tegument proteins and secreted peptides were mapped to the *S*. *bovis* genome using Exonerate [54]. The identified *S*. *bovis* orthologous were then mapped to *S*. *haematobium*, again using Exonerate using the options—model protein2genome—proteinwordlen 5—minintron 15—score 80—percent 50.

### Comparative analysis

*S*. *bovis* homologs were inferred from the genomes of the three *Schistosoma* species using Exonerate, v.2.4.0 [54]. Matches with a sequence identity below 60% or that spanned less than 40% of the query protein were excluded. Exonerate was also employed for mapping known vaccine targets and cancer-associated proteins to the *S*. *bovis* genome sequence.

The genome sequences of *S*. *bovis*, *S*. *haematobium*, *S*. *mansoni* and *S*. *japonicum* were compared based on pairwise whole-genome alignments using Progressive-Mauve, 2015-02-13 snapshot [55]. Repeats were masked prior to the alignment step to minimize alignment artefacts. The sequence identity across the resulting alignments were analyzed in 5 kb windows, including only those windows with at least 2 kb of aligned sequence (de-gapped and excluding Ns).

Several regions with high sequence similarity discovered from the *S*. *bovis-S*. *haematobium* whole-genome alignment were investigated further by first carrying out a 3-way alignment of *S*. *bovis*, *S*. *haematobium* and *S*. *mansoni* using Progressive-Mauve, ordering the aligned segments according to the *S*. *mansoni* reference genome. Sequence identity across the alignments were analyzed in 5 kb windows. We then segmented the genome into blocks with similar degrees of sequence similarity by fitting a piecewise constant curve using a least squares cost function as implemented in the segment function of the tilingArray R package [56]. For selected loci, the corresponding un-masked genome sequences were obtained for *S*. *bovis* and *S*. *haematobium* and arranged in the order of the *mansoni*-guided 3-way alignment. Pairwise alignments with mafft, v6.603b, with the–auto option [57] were then carried out separately for each region, and the sequence identity calculated for 1 kb windows across the resulting alignments.

For the identification of genomic mutations and structural variations, *S*. *bovis* paired-end sequence data were mapped to the *S*. *haematobium* genome using BWA-MEM, v.0.7.12 [58], and PCR duplicates were marked using Picard’s MarkDuplicates, v.2.2.1. GATK, v.3.5, [23] was employed for locally realigning reads adjacent to indels and for base quality score recalibration. Single nucleotide substitutions (SNSs) and short indels were then called using the GATK UnifiedGenotyper framework, requiring at least 10 reads to support a variant. Functional effects were annotated with SnpEff, v.4.2 [59]. Structural variations, such as translocations, inversions and long indels were called from the aligned paired-end sequence data using BreakDancer, v.1.1 [60]. Identified SNSs, indels and structural variations were filtered using in-house Python scripts. Predicted structural variations were manually verified in the Integrative Genomics Viewer (IGV) [61] based on the generated paired-end Illumina sequence data.

To infer a *Schistosoma* pan-genome, the concatenated proteomes of *S*. *bovis*, *S*. *haematobium*, *S*. *mansoni* and *S*. *japonicum* were clustered into orthologous groups using PISCES, v.1.0 [53], with a 60% sequence-identity cut-off (-p 60). Exonerate, v.2.4.0 [54], was then employed to map orthologous groups to each *Schistosoma* genome (options:—model protein2genome—proteinwordlen 5—minintron 15—score 80—percent 50). Matches with a sequence identity below 50% or that spanned less than 25% of the query protein were excluded.

### Population analysis

For the identification of polymorphisms in *S*. *bovis* and *S*. *haematobium* whole genome and targeted exome libraries, the raw reads from the *S*. *bovis* 200 bp library, and the *S*. *haematobium* libraries listed under “Data” were mapped to the *S*. *bovis* and *S*. *haematobium* genomes and variants were identified with the GATK UnifiedGenotyper framework [23], as described above. To facilitate the characterisation of shared variants all the mapped libraries were used as input for UnifiedGenotyper and filtered with the GATK VariantFiltration feature using the options "DP<15" and "MQ<30.0.

### Phylogenetic analysis

We reconstructed a phylogenetic tree using protein sequences inferred from the genomes of *S*. *bovis*, 12 selected fluke species and *Schmidtea mediterranea* as an outgroup. Single-copy genes were first identified by matching proteins against the genome of the same species using blastp, v.2.2.30 [62]. Matches with sequence identity >30% and aligned sequence coverage >50% were excluded. Single-copy genes present across all included species were identified using blastp with a sequence identity cut-off of 40% and requiring coverage >40%, resulting in 52 shared single-copy genes **(S1 Data)**. The resultant proteins were concatenated into a single sequence and aligned with MUSCLE, v.3.8.425 [63], and the resultant alignment was de-gapped with trimAl [64] and manually checked. The final alignment had 9,341 aligned positions that were then used to reconstruct a phylogenetic tree using PROML within Phylip v.3.696 [65] and the Jones-Taylor-Thornton substitution model (options: JTT probability model, search for best tree, one category of sites, constant rate of change, un-weighted sites and *S*. *mediterranea* as outgroup). Using the generated topology as input, species divergence was estimated by a Bayesian relaxed molecular clock model using MCMCTREE in PAML v.4.9e [66]. The model was calibrated using previously published divergence times and ages of fossil records of intermediate snail hosts. MCMCTREE was run with options seed = -1, ndata = 1, seqtype = 2, usedata = 3, clock = 2, RootAge = '<4.44', model = 0, alpha = 0, ncatG = 5, cleandata = 0, BDparas = 1 1 0.1, kappa_gamma = 6 2, alpha_gamma = 1 1, rgene_gamma = 2 20 1, sigma2_gamma = 1 10 1, finetune = 1: .1 .1 .1 .1 .1 .1, print = 1, burnin = 2000, sampfreq = 10 and nsample = 20000.

### Declarations

#### Availability of data and materials

The nuclear and mitochondrial genome assemblies are available in the NCBI repository PRJNA451066.

## Supporting information

S1 Text Supporting results, figures and tables. Supporting results, Figures A-K with corresponding legends and Tables A-F.
(PDF)

S1 Data Phylogeny_protein_sequences.tar.gz. (TGZ)

S2 Data Mitochodrial_repeats.fa.gz. (GZ)
