# Supplementary material for: Whole-genome sequence of the bovine blood fluke Schistosoma bovis supports interspecific hybridization with S. haematobium
Source: PLoS Pathog. 2019 Jan 23;15(1):e1007513. doi: 10.1371/journal.ppat.1007513 (PMC6361461; doi:10.1371/journal.ppat.1007513)
Supplement: S1 Text — Supporting results, Figures A-K with corresponding legends and Tables A-F. (PDF) [file ppat.1007513.s001.pdf]

Supplementary Material

Whole-genome sequence of the bovine blood fluke *Schistosoma bovis* supports interspecific hybridization with *S. haematobium*

Harald Oey[1], Martha Zakrzewski[2], Kerstin Gravermann[5], Neil D. Young[4], Pasi K. Korhonen[4], Geoffrey N. Gobert[2,3], Sujeevi Nawaratna[2], Shihab Hasan[1,2], David M. Martinez[2], Hong You[2], Martin Lavin[9], Malcolm Jones[2,8], Mark A. Ragan[6], Jens Stoye[5], Ana Oleaga[10], Aidan Emery[7], Bonnie Webster[7], David Rollinson[7], Robin B. Gasser[4], Donald P. McManus[2,*], Lutz Krause[1,2,*,#]

Supplementary Results

Vaccine Targets

Schistosomes have been targeted for elimination by the World Health Organization, but despite global efforts to reduce disease infection, rates in ruminants and humans continue to be high in most endemic regions. No *Schistosoma* vaccines are currently available, and treatment relies on one drug, praziquantel [1]. Praziquantel chemotherapy is highly effective but even mass treatment does not prevent reinfection. Vaccines that induce long-term immunity represent a logical component for the future control of schistosomiasis with the final goal of complete elimination. Currently, only three schistosome vaccine antigens have entered human clinical trials. Two recombinant *S. mansoni* vaccines are in early stage clinical testing (Sm14 and Sm-TSP-2) and one *S. haematobium* specific vaccine (Sh28GST or Bilhvax) completed a Phase 3 trial [2, 3]. The *S. mansoni* molecule Smp80 (calpain) is undergoing testing in non-human primates and will likely advance to clinical development [3]. We identified highly conserved *S. bovis* orthologous of Sm14 (99% identify), Sh28GST (97.2%) and Sm-p80 (98.5%), and a moderately conserved orthologue of the tetraspanin Sm-TSP-2 (34%) (**Table S5**). Tetraspanins are composed of two domains, the short EC1 domain and longer EC2 domain. All four included schistosome species were conserved over the EC1 domain (65% identity), but showed two different variants of the EC2 domain. These results indicate that *Schistosoma* vaccine targets potentially show cross-reactivity for multiple *Schistosoma* species and represent promising vaccine target candidates against *S. bovis*.

Supplementary Figures

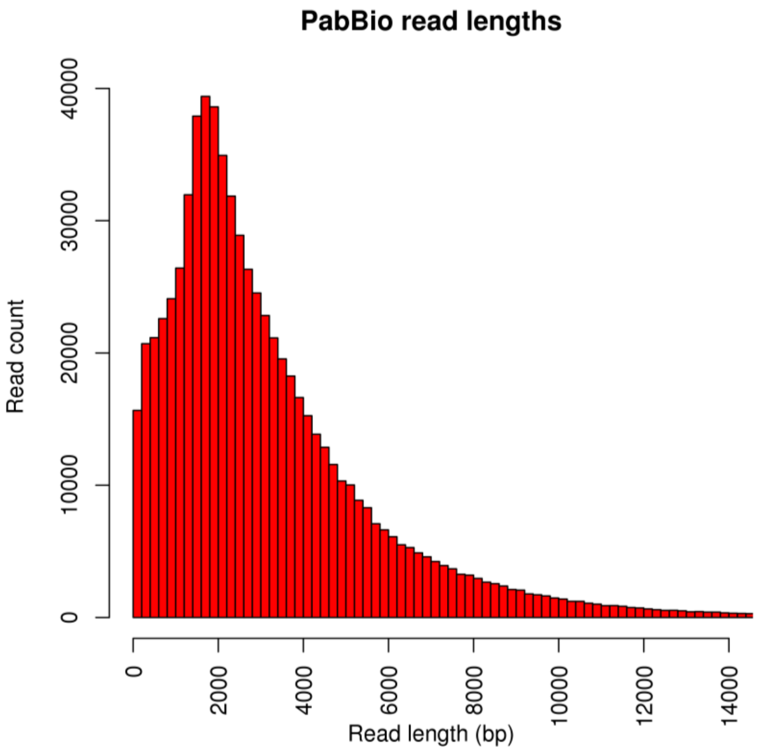

Figure A: PacBio read length distribution

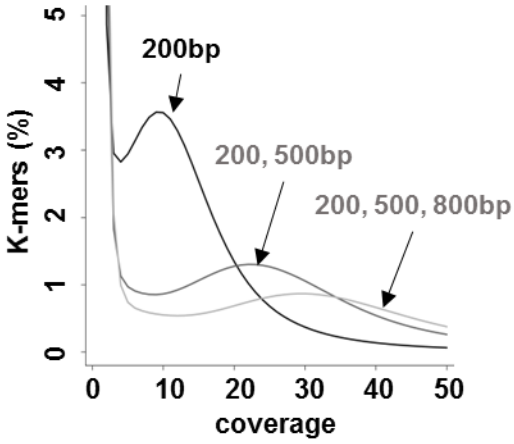

Figure B: Distribution of 17-mers in short insert Illumina libraries.

The x-axis represents k-mer coverage (17 base pairs), the y-axis the distribution (percentage) of k-mers with that coverage. The peak at 10 indicates that 3.5% of all detected 17-mers occurred about 10 times in the 200bp insert libraries. The frequency analysis demonstrated a low sequence heterozygosity of *S. bovis*.

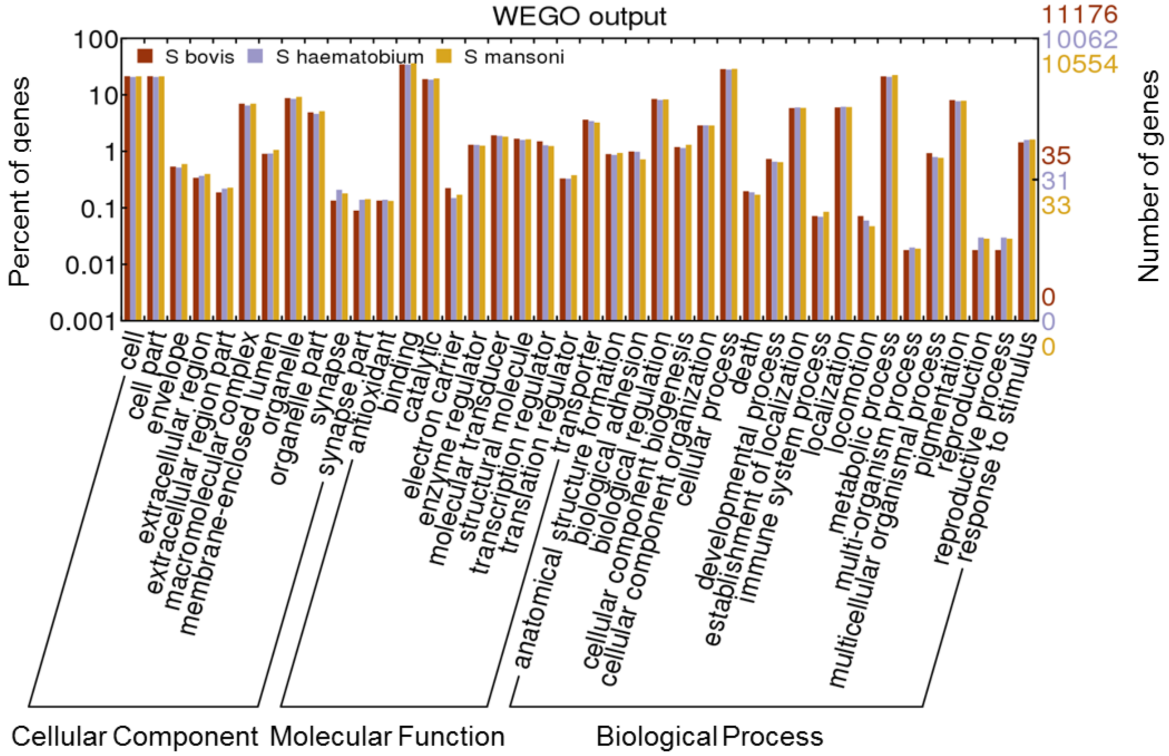

Figure C: GO categories identified across three *Schistosoma* species.

GO annotations were assigned from InterProScan results using WEGO.

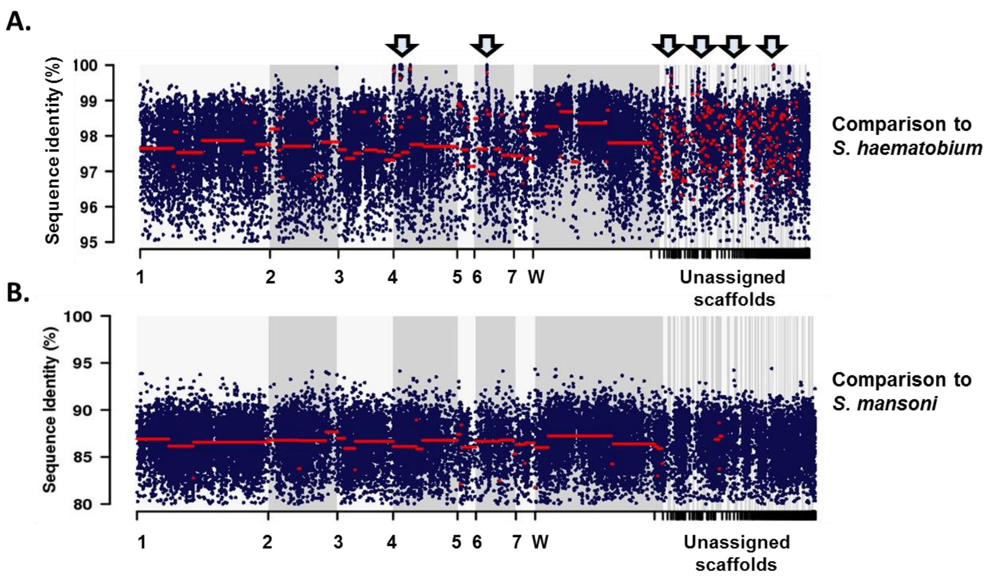

Figure D: Genome-wide comparison of *S. bovis* with *S. haematobium* and *S. mansoni*.

Genome-wide comparison of *S. bovis* and *S. haematobium,* panel (A), and *S. bovis* and *S. mansoni,* panel (B). Genomic similarity was measured in a sliding-window approach using a 5kb window size. Chromosome numbers were assigned by mapping *S. bovis* scaffolds to the *S. mansoni* genome assembly (autosomal chromosomes 1-7 and sex chromosome W). Several long contiguous and highly conserved regions (sequence-identity > 99%) provide evidence of recent inter-species hybridization and are marked with arrows. Red lines depict segments with similar level of similarity, which were found by fitting a piecewise constant curve using a least squares cost function.

A)

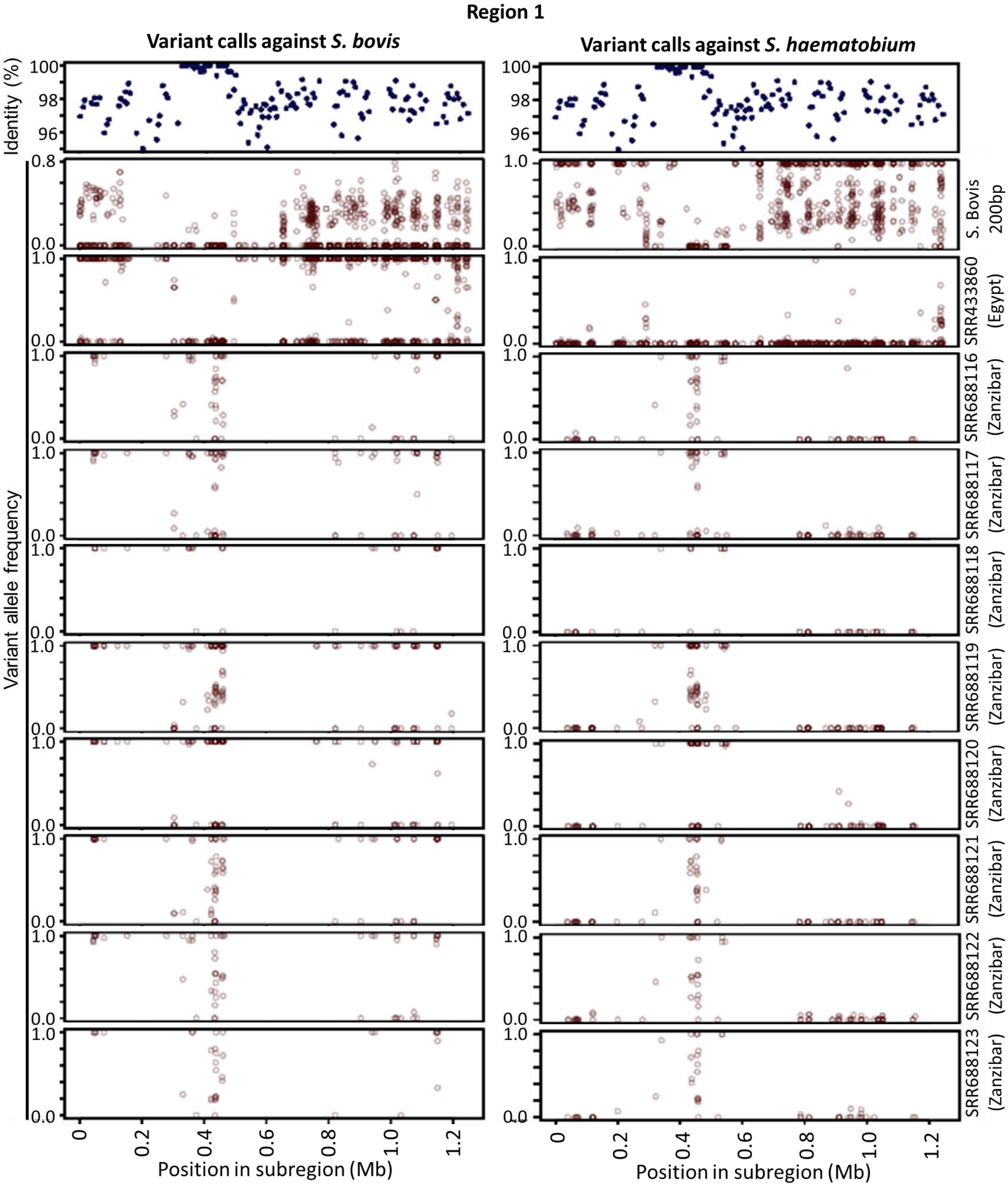

Region 1
Variant calls against S. bovis
Variant calls against S. haematobium
Identity (%)
100
98
96
Variant allele frequency
0.8
1.0
0.0
S. Bovis 200bp
SRR433860 (Egypt)
SRR688116 (Zanzibar)
SRR688117 (Zanzibar)
SRR688118 (Zanzibar)
SRR688119 (Zanzibar)
SRR688120 (Zanzibar)
SRR688121 (Zanzibar)
SRR688122 (Zanzibar)
SRR688123 (Zanzibar)
0
0.2
0.4
0.6
0.8
1.0
1.2
Position in subregion (Mb)

B)

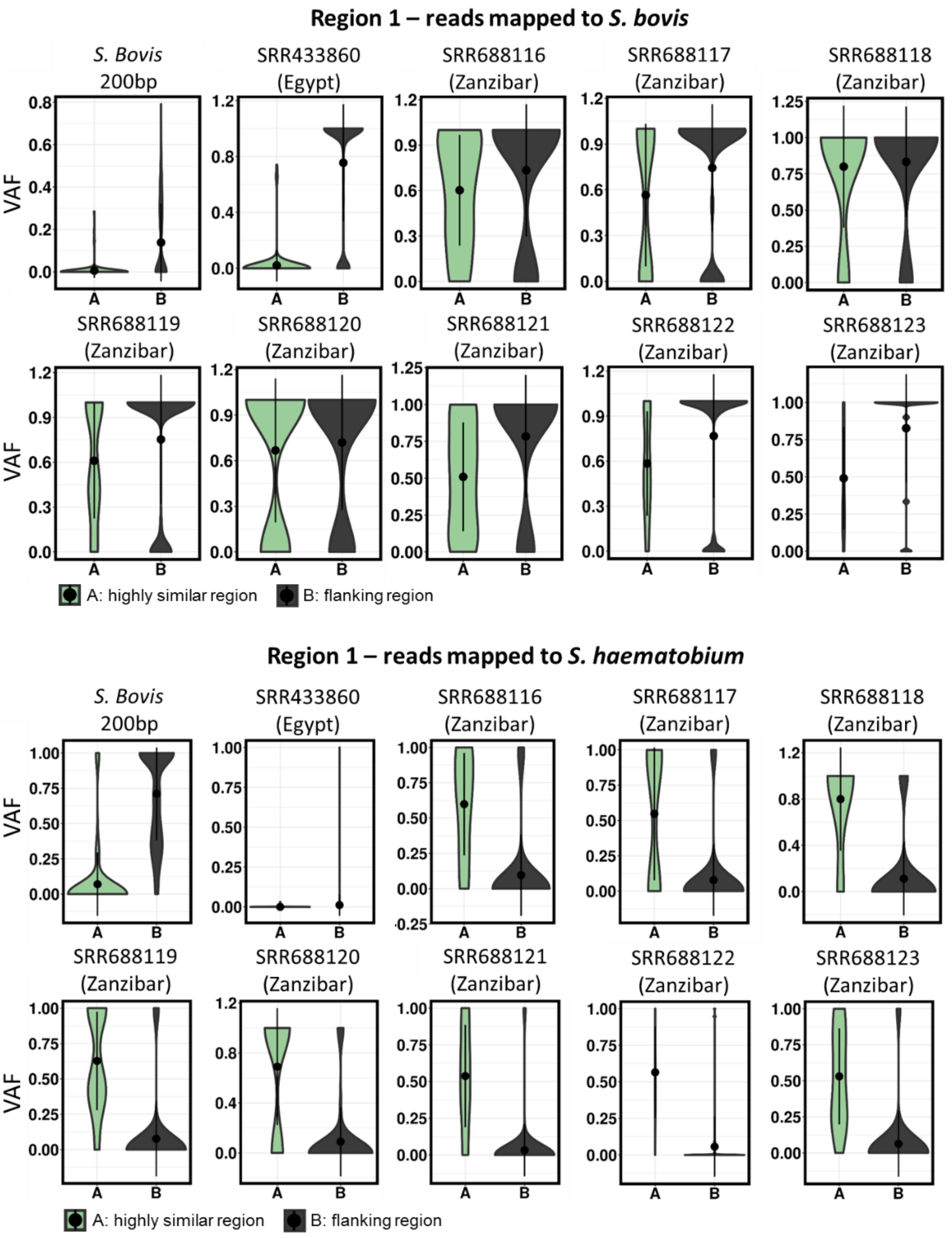

Figure E: Variant allele frequencies for *S. bovis* and *S. haematobium* polymorphisms for region 1

Panel (A) The figure shows the sequence identity scores for region 1, and the flanking regions (top panel) and the variant allele frequencies of variants identified by mapping whole genome sequencing reads (*S. bovis* and *S. haematobium,* Egypt isolate) and exome reads (*S. haematobium,* Zanzibar isolates) to either the *S. bovis* or the *S. haematobium* genome assemblies. Panel (B) Violin plots of the variant allele frequency distributions for variants within and flanking the highly similar region, as indicated.

A)

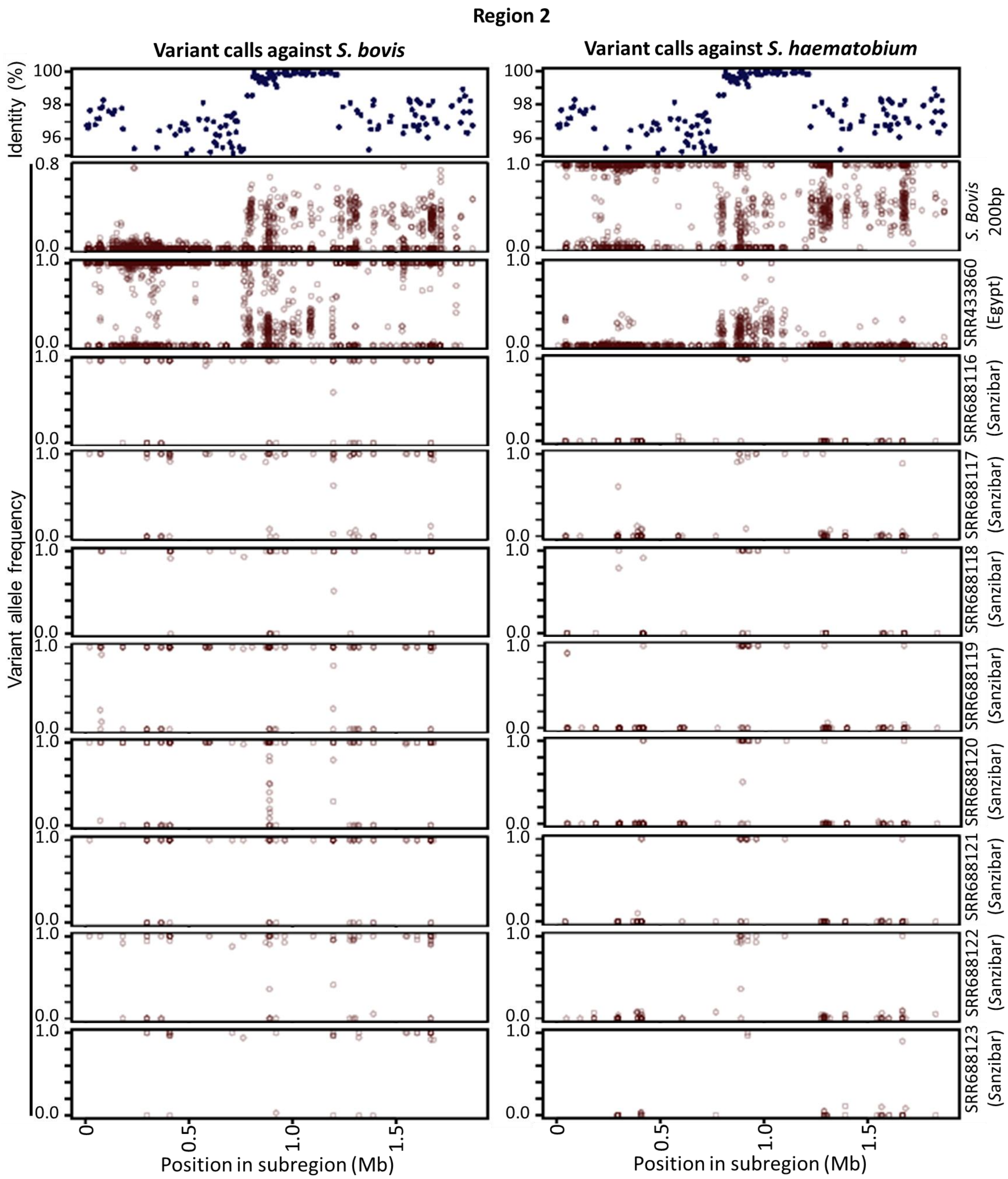

Region 2
Variant calls against *S. bovis*
Variant calls against *S. haematobium*
Identity (%)
100
98
96
Variant allele frequency
0.8
0.0
1.0
S. Bovis 200bp
SRR433860 (Egypt)
SRR688116 (Sanzibar)
SRR688117 (Sanzibar)
SRR688118 (Sanzibar)
SRR688119 (Sanzibar)
SRR688120 (Sanzibar)
SRR688121 (Sanzibar)
SRR688122 (Sanzibar)
SRR688123 (Sanzibar)
0
0.5
1.0
1.5
Position in subregion (Mb)

B)

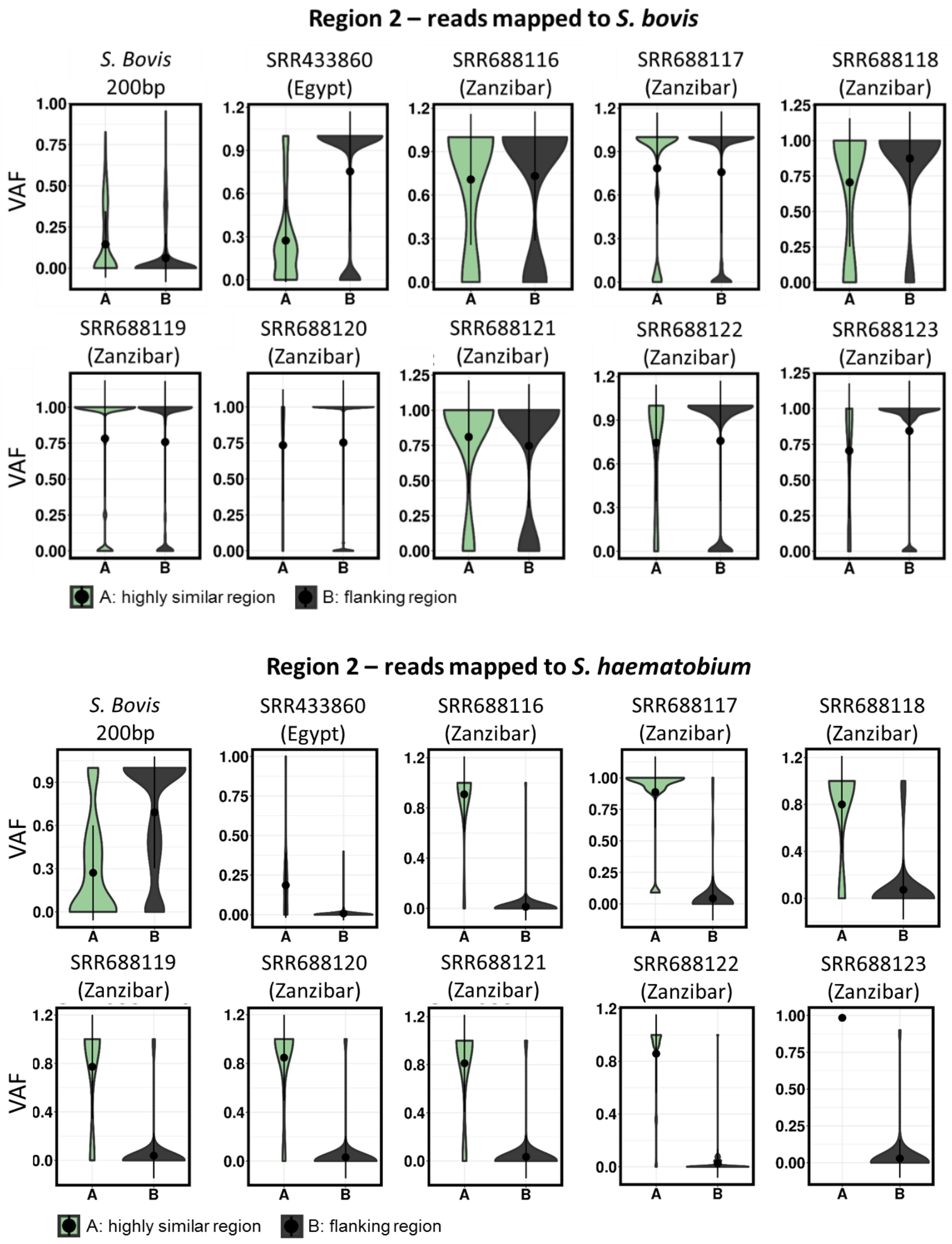

Figure F: Variant allele frequencies for *S. bovis* and *S. haematobium* polymorphisms for region 2

Panel (A) The figure shows the sequence identity scores for region 2, and the flanking regions (top panel) and the variant allele frequencies of variants identified by mapping whole genome sequencing reads (*S. bovis* and *S. haematobium,* Egypt isolate) and exome reads (*S. haematobium,* Zanzibar isolates) to either the *S. bovis* or the *S. haematobium* genome assemblies. Panel (B) Violin plots of the variant allele frequency distributions for variants within and flanking the highly similar region, as indicated.

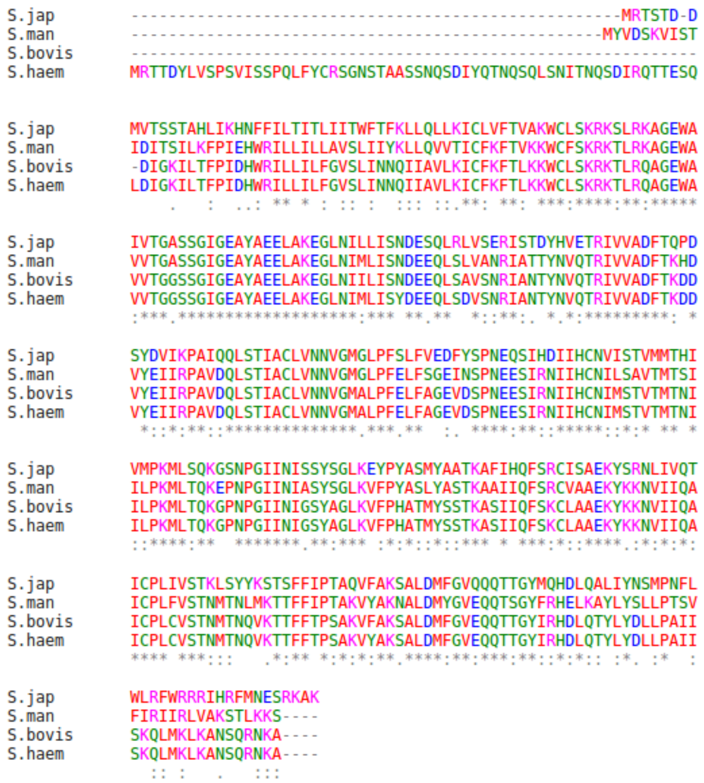

Figure G: Conservation of estradiol 17beta-dehydrogenase across multiple *Schistosoma* species.

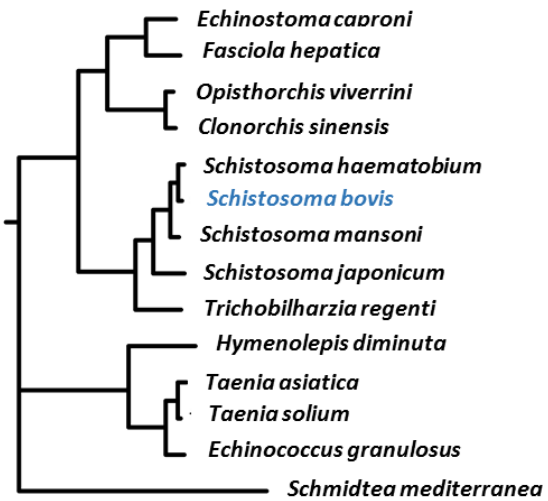

Figure H: Phylogenetic tree reconstructed from the amino acid sequences of 52 shared single-copy genes using the maximum-likelihood method.

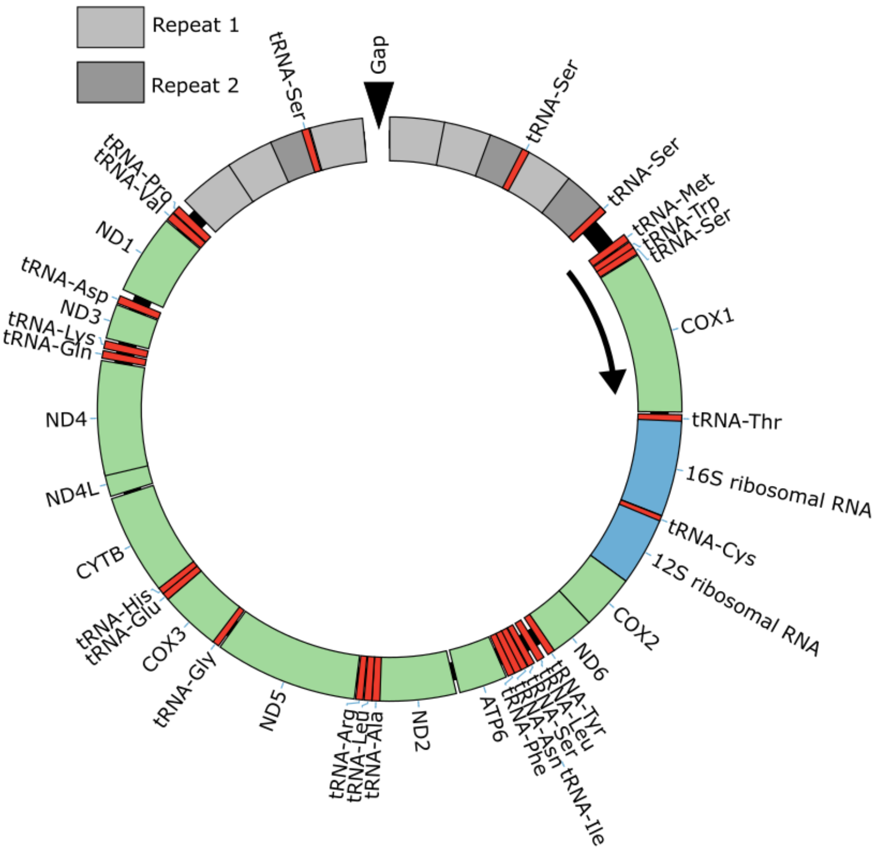

Figure I: Circular genome plot of the *S. bovis* mitochondrial genome.

The mitochondrial genome sequence was *de-novo* assembled from long PacBio sequence reads yielding a 20 kb contig. Shown are predicted proteins (green), rRNAs (blue) and tRNAs (red). All predicted genes are transcribed in the same direction, as indicated by the black arrow. A >4 kb repetitive region comprised of two distinct repeat units is depicted in grey. Repeat unit 1 (light grey) has ~520 bp; unit 2 (dark grey) has ~460 bp and contains a predicted tRNA for the amino acid serine. The repetitive region was not completely resolved in the assembly and the position of the gap is indicated at the top of the circle.

```
PacBio_read      TATTCTACGAAACTAACTCCTACTCTCTTATCAAAACTTTATA-AGTCAGAACCTCTGTA
SB_Mito_rep      --------ATAAGTATGTTTAGCGCTATCAT----AGTTTATATAGTTA----------A
                           ** **  *    * ** * **    * ****** *** *          *

PacBio_read      TAAACCCAACAATACCAACCTTTTGGCCCCTTACGTAATTGAATATAGGATAAGACCTTA
SB_Mito_rep      TAAAGCT-------TTAATTTTTTGGTTTTTTTATGAATT--------------ATCTAT
                 **** *          **  ******    **    ****              * **

PacBio_read      CGTTCTCCCATTATATA--------AAAGGCTACTAATAATAAAATCATTATTAAACT--
SB_Mito_rep      TATTTGTGCATTATACATGTTGCTTAAGGAGAGTTTATACCCAGAGCGTTATAAAGCTAG
                   **    ******* *        ** *     * ***   * * * **** ** **

PacBio_read      --------------------CTCAATACTCAAAAATAACTC-TAACATATACATAT----
SB_Mito_rep      GTGATATATGGGTGGGTATGTTC--TTCTTAGAAGTGTTCCATTACTTATATGTATTTTG
                                      **  * ** * ** *    * * ** ****  ***

PacBio_read      -CCGTA--ATTCGCTCAA---------AATATCTACGCCTGATAAACCAAACACTATTA-
SB_Mito_rep      ATTGTAGGGTTAGTTGAGGGTAGGTTTAATATTTTAGCTTGAGATAATAAA-AGTATGAA
                    ***   ** * * *          ***** *  ** *** * *  *** * *** *

PacBio_read      -------TCAATCTACTAA--AATTATTAAAATAATTTTTA--AATAATCCA--------
SB_Mito_rep      TTTGAGTTTAGTTTATGTAGTAATGATTATAATGTTTTGTACGAAGATCCCATTGATTCC
                        * * * **   *  *** **** ***  *** **  ** *  ***

PacBio_read      ----CATACACTGACCATAAAT-----ATACTAATCACCAAAAGCACGACAAGCTTCTAT
SB_Mito_rep      GTTTCATAGA-TGACTACCAATGGTTCATGCTGA-AGCTAGTAGTCCTACAAGTATA-AT
                     **** * **** *  ***     ** ** *   * *  **  * *****  *  **

PacBio_read      CTTCATAAACTAAATTAT------------------TATCAACCATATGC---CCACATA
SB_Mito_rep      ATTAAGAGGGTATATTATGAAGTTGGGTTTAGTCGGTGTTGTCCGTTTATGTGGTAGATG
                  ** * *   ** *****                  * *   ** * *       * **

PacBio_read      CACATAATTACTATTATTTAATCCACTTTA------TATACCCCAACTTAAGCTCTGCCA
SB_Mito_rep      AGTATGAGGGTTAGGAATAAATAGAGTTTCAGAGTTTATATTTTTGTTAAGGTTAGGATT
                    ** *    **  * * ***  * ***       ****       * * * *  *

PacBio_read      TATAACCAAGAACCAAAAAAAAAAAAAAAAAAAAAATAACACCAAAAGGTGTAA----TATC
SB_Mito_rep      TTTGATTTGGGCTTATTATGAGGTTGATTCAAAGCGTTGACTGGCTGTGTTAAGTCTATC
                 * * *    *    *  *  *     *   ***       *     **** *    ****

PacBio_read      ATATACCGTCATAAACTATATTTAATAA---TAATGATATTTCTAAATCGAA--------
SB_Mito_rep      TCATATAATGGTTAGAGTTATTTTATTAGTTTATGGGTGTTACGATGTTAAATTATTGGC
                   ***   *  * *    ***** ** *   **  * * ** * *  *  **

PacBio_read      ----------------GACCACAAATA---------------ATATTAATAGATTTAAAT
SB_Mito_rep      CTATATCTTTTGTCTTGGTCATGGTTATTCTGTGTGTGGTATGTTTTTGTTAATTTGATG
                                 *  **    **                * **  *  **** *

PacBio_read      AAG------------AAAAACTAAAAAATTTTTCATTCAACCCATGCCCTAAAAACCCAC
SB_Mito_rep      AGGCTATAATCATGTGAAATCTCGGAGATGATTGATTTTAACTAAGTTTTACAGCCTTGT
                 * *             *** **   * **  ** ***  * * * *   ** *  *

PacBio_read      ATTCGAATCTTGAATATCAT---TCTGACATGTTACTATAACGATTAC----CTTCGAAA
SB_Mito_rep      TCCTGTAATTCAAGTGTTATGTGTTTTA-ATGTT--TATGAGGGTTTCAAGTTTTCCACC
                     * *  *  * * * **   * * * *****  *** * * ** *     *** *

PacBio_read      TGCTTTACCCCAAAAACACTATAGTGAACTAACCCTACCAATAATAACACTGAAAATATA
SB_Mito_rep      TACTTTGC------AATTTTTTAGTGAGGTGATACTAGC-------ATGGTGCAGAT-TG
                 * **** *      **   * ******  * *  *** *       *   ** * ** *

PacBio_read      ACTGTTAATAATACT------AAACTCAGTTTAGACACTTTATTACTCGGCCA-----CG
SB_Mito_rep      GCTGTTAATAATGATATATTGACATTTATTATATGTATCTATTTATTTGGTAGTAGATTG
                  ***********  *      * * * * * **   *  *  *** * **         *

PacBio_read      ATATGTTTAATTATAAT----------CTAACTTAGCT-----ATGCTAATAG----GTT
SB_Mito_rep      GTAGGGTTAATGGTATTAGGATTAGTCTTAATTTACTTGTTAGATTATAGTAGTATTGTT
                  ** * *****  ** *           *** ***  *     **  ** ***    ***

PacBio_read      CATATCTATAACATACTAAGATTAAGAGT-CTTATGCTTTA------ATTCTTA-----A
SB_Mito_rep      CATTACAATAATAGAAAAAGTTTAGGTGTAAGTATAATTTATATAGGAATCTTAATGTTA
                 ***  * **** * *  *** *** * **   ***  ****      * *****     *

PacBio_read      GCTACTATCAGTAAAATCTATTATTGCTAACTACAATCATCTTATGTTGTATGTAAAAAC
SB_Mito_rep      TTTACTTTTATTGTGGTTTAGTTATGTTATAAATATTTGGGTAAAGTTGTA-GTAGTAAC
                   **** * * *    * ** *  ** **   * * *    * * ****** ***  ***

PacBio_read      TGGCATTAA----------CTAAACCACCT----CTTTGACCAAAACAAAACATGCTATA
```

```
SB_Mito_rep      -GATGTTAAAAACTTAGTGTTATAGCATGTTTTGTTTTGGTCAAAGAGGTGGTTTAGTTA
                  *   ****           ** * **  *     ****  ****        *    **

PacBio_read      ACACTAAGTTTTTAACATCGTTACTCTACAACAT-AGATGATTTAGTGTTAGCAATAA-A
SB_Mito_rep      ATGCC-AGTTTTTA----------CATACAACATAAGATGA-TTGTAGTTAGCAATAATA
                 *  *  ********            ******** ****** **   *********** *

PacBio_read      GATTTTACTGATAGTAGCTTAAGAAT--AAGCATAAGGACTCTTAATC-TAGTATGTTAT
SB_Mito_rep      GATTTTACTGATAGTAGCTTAAGAATTAAAGCATAA-GACTCTTAATCTTAGTATGTTAT
                 **************************  ******** *********** ***********

PacBio_read      AAGATATGAACCTATTAGCATAGCTAAGT--GTTAATAATT-AACATATCGTGGCCGAGT
SB_Mito_rep      -AGATATGAACCTATTAGCATAGCTAAGTTAGATTATAATTAAACATATCGTGGCCGAGT
                  ****************************  * * ****** ******************

PacBio_read      AATAAA--GGTTAAACTGAGTTTAGTATTATTAAC---TATATTTTTCAGTGTTATTATT
SB_Mito_rep      AATAAAGTGTCTAAACTGAGTTTAGTATTATTAACAGTTATA-TTTTCAGTGTTATTATT
                 ******  *  ************************   **** *****************

PacBio_read      GGTAGGGTTAGTTCACTATAGTGTTTTTGGGGTAAAGCATTTCGAAGGTAATCTTTATAG
SB_Mito_rep      GGTAGGGTTAGTTCACTATAGTGTTTTTGGGGTAAAGCATTTCGAAGGTAATCGTTATAG
                 ***************************************************** ******

PacBio_read      TAACATG-CAGAATGATATTCAAGATTCG-ATGTGGGTTTTTAGGGCATGGGTTGAATGA
SB_Mito_rep      TAACATGTCAGAATGATATTCAAGATTCGAATGTGGGTTTTTAGGGCATGGGTTGAATGA
                 ******* ********************* ******************************

PacBio_read      AAAATTTTTTAG-TTTTCTTATTTAAATCTATTAATATTATTTGTGGTCTTCGATTTAGA
SB_Mito_rep      AAAATTTTTTAGTTTTTCTTATTTAAATCTATTAATATTATTTGTGGTCTTCGATTTAGA
                 ************ ***********************************************

PacBio_read      AATATCATTATTATTAAATATAGTTTATGACGGTATATGATATTACACCTTTTGGT----
SB_Mito_rep      AATATCATTATTATTAAATATAGTTTATGACGGTATATGATATTACACCTTTTGGTGTTA
                 ********************************************************

PacBio_read      TGTTATTTTTTTTTTTTTTTTGGTTCTTGGTT-TATGGCAGAGCTTAAGTTGGGGTATAT
SB_Mito_rep      TTTTTTTTTTTTTTTTTTTTTGGTTCTTGGTTATATGGCAGAGCTTAAGTTGGGGTATAT
                 * ** *************************** ***************************

PacBio_read      AAAGTGGATGTA---------AATTATGTGTATGTGGGCATATGGTTGATATACATTT-G
SB_Mito_rep      AAAGTGGATTAAATAGATAGTAATTATGTGTATGTGGGCATATGGTTGATATACATTTAG
                 *********  *         ************************************* *

PacBio_read      TTTATGAAGAATAGAAGCTTGTCGTGCTTTTGGTGATTAGTATATTTATGGTCAGTGTAT
SB_Mito_rep      TTTATGAAGAATAGAAGCTTGTCGTGCTTTTGGTGATTAGTATATTTATGGTCAGTGTAT
                 ************************************************************

PacBio_read      GTGGATTATTTAAAA--ATATTTTAATAATTTTAGTA----GATGATATAGTGTTTGGTT
SB_Mito_rep      GTGGATTATTTAAAAATATATTTTAATAATTTTAGTAGATTGATAATATAGTGTTTGGTT
                 ***************  ********************    *** ***************

PacBio_read      TATCAGGCGTAG--ATTTTGAGCGAATTACGGATATGTATATGTTAGAGTTATTTTTGAG
SB_Mito_rep      TATCAGGCGTAGATATTTTGAGCGAATTACGGATATGTATATGTTAGAGTTATTTTTGAG
                 ************  **********************************************

PacBio_read      TATTGAGAGTTTAATAATGATTTTATTATTAGTAGCCTTTTATATAAT-GGAGAACGTAA
SB_Mito_rep      TATTGAGAGTTTAATAATGATTTTATTATTAGTAGCCTTTTATATAATGGGAGAACGTAA
                 ************************************************ ***********

PacBio_read      GGTCTTATCCTAT-TTCAATTACGTAAGGGGCCTAAAAAGGTTGGTATTGTTGGGTTACT
SB_Mito_rep      GGTCTTATCCTATATTCAATTACGTAAGGGGCCTAAAAAGGTTGGTATTGTTGGGTTACT
                 ************* **********************************************

PacBio_read      ACAGAGGTTTGCTGACTTTATAAAGTTGTTAAGTAAGAGTAAGTTAGGAGAGTTTAGGTT
SB_Mito_rep      ACAGAGGTTTGCTGACTTTATAAAGTTGTTAAGTAAGAGTAAGTTAGGAGAGTTTAGGTT
                 ************************************************************

PacBio_read      TCGTAGATGATTTTCATTGTTAGGTTGCGTAATTTTAGTTTCATGCTCTTTGTCAATGGT
SB_Mito_rep      TCGTAGATGATTTTCATTGTTAGGTTGCGTAATTTTAGTTTCATGCTCTTTGTCAATGGT
                 ************************************************************

PacBio_read      GGTTATTTATAGATTAATAAATAGGAAAATGTGTAGGAAATGAATATTACTTTACCTTTT
SB_Mito_rep      GGTTATTTATAGATTAATAAATAGGAAAATGTGTAGGAAATGAATATTACTTTACCTTTT
                 ************************************************************

PacBio_read      AGTAGTATGTTCTGTCTTAAGTTATAGA--CTTATTGATAGGTTGATGTTCATGGTCTAA
SB_Mito_rep      AGTAGTATGTTCTGTCTTAAGTTATAGAACTTATTGATAGGTTGATGTTCATGGTCTAA
```

```
                           ****************************   *****************************

PacBio_read                GTATGGTTTAATTAGTTCTATTCGTGTATCCTTTTCTAGTGTGATGTTTGAGATGATACT
SB_Mito_rep                GTATGGTTTAATTAGTTCTATTCGTGTATCCTTTTCTAGTGTGATGTTTGAGATGATACT
                           ************************************************************

PacBio_read                GATGTGTATAATAATTCTGTTTGGTTTAATGTACGGTAGCTATGGGAAACCTGAC----A
SB_Mito_rep                GATGTGTATAATAATTCTGTTTGGTTTAATGTACGGTAGCTATGGGAAACCTGACATTAA
                           *******************************************************    *

PacBio_read                TAGAA-TATGATATTTGTAGTACCTCTGGTTTACATCATATGACTTATAGTGTTACTAAG
SB_Mito_rep                TAGAATTATGATATTTGTAGTACCTCTGGTTTACATCATATGACTTATAGTGTTACTAAG
                           ***** ******************************************************

PacBio_read                TGAGAGTAATCGAACACCTTGTGATTATGTAGAGTCCGAGAGTGAATTAGTAAGAGGGAT
SB_Mito_rep                TGAGAGTAATCGAACACCTTGTGATTATGTAGAGTCCGAGAGTGAATTAGTAAGAGGGAT
                           ************************************************************

PacBio_read                TAGAGTAGAATATAGGAGTA-TTTTTTTTTAATTATATTTGCTTGTGAATATTTAATTAT
SB_Mito_rep                TAGAGTAGAATATAGGAGTATTTTTTTTTTAATTATATTTGCTTGTGAATATTTAATTAT
                           ******************** ***************************************

PacBio_read                GTTTATATTTAGTTGAGTGAGGTTTACGATATTTTGAAGTATAAATGAGATGTTAATAGT
SB_Mito_rep                GTTTATATTTAGTTGAGTGAGGTTTACGATATTTTGAAGTATAAATGAGATGTTAATAGT
                           ************************************************************

PacBio_read                AATGAATTTGATGCTGTTTATAATGATGCGTGGTTCGTTTCCTCGACTTCGGTTTGATGT
SB_Mito_rep                AATGAATTTGATGCTGTTTATAATGATGCGTGGTTCGTTTCCTCGACTTCGGTTTGATGT
                           ************************************************************

PacBio_read                ATTTGTTTCAGTGATTTGAGAGTATTGTGTTATGGTAATATTGATATATTTAATATGTTT
SB_Mito_rep                ATTTGTTTCAGTGATTTGAGAGTATTGTGTTATGGTAATATTGATATATTTAATATGTTT
                           ************************************************************

PacBio_read                ATTTAGAATATTATAATGAGTCTATTTTAGTATCTGTAGTAAAATTGATTAGCGTAGTAG
SB_Mito_rep                ATTTAGAATATTATAATGAGTCTATTTTAGTATCTGTAGTAAAATTGATTAGCGTAGTAG
                           ************************************************************

PacBio_read                TTAAAGTTTAACGTTTG-TTTACACACAAAAGATGAAGTAAGGTTTGAGTATGCTTCCGG
SB_Mito_rep                TTAAAGTTTAACGTTTGTTTTACACACAAAAGATGAAGTAAGGTTTGAGTATGCTTCCGG
                           ***************** ******************************************

PacBio_read                CGCCATAATTATTTTTTTAAATTTAGTTTGATCAGTTAAGTAGTTTGTATAAAGAATATA
SB_Mito_rep                CGCCATAATTATTTTTTTAAATTTAGTTTGATCAGTTAAGTAGTTTGTATAAAGAATATA
                           ************************************************************

PacBio_read                AGCTTTGGGTGCTTAAGGATCTATTTATTAAAGTAGACTTACTGAGGTTATAGGTTAAAG
SB_Mito_rep                AGCTTTGGGTGCTTAAGGATCTATTTATTAAAGTAGACTTACTGAGGTTATAGGTTAAAG
                           ************************************************************

PacBio_read                TAGTAAAAAGTATATAAGGCTTATAGTAAGCTTCACCATCGAATGGTGAGTAAGATTTAA
SB_Mito_rep                TAGTAAAAAGTATATAAGGCTTATAGTAAGCTTCACCATCGAATGGTGAGTAAGATTTAA
                           ************************************************************

PacBio_read                AA---TCTCTTTATAGTC------TTGTTTTTGTTTTTTTTTTGGTGGTGGTAAGTTTAG
SB_Mito_rep                AAATCTCTATTTATAGTCTTGTTTTGTTTTTGTTTTTTTTTTGGTGGTGGTAAGTTTAG
                           **   *** *********      ************************************

PacBio_read                TTGTATTTTAGTTCCAATAAAAATTTTTTGTAAAAAGTATGTTTTAATTCATTTATATAT
SB_Mito_rep                TTGTATTTTAGTTCCAATAAAAATTTTTTGTAAAAAGTATGTTTTAATTCATTTATATAT
                           ************************************************************

PacBio_read                AATAT----TTATATAATATAATATAATGTAAAATAAGTATAGTATAGTA-GGGGGAAAA
SB_Mito_rep                AATATATTATAATATAATATAATATAATGTAAAATAAGTATAGTATAGTAGGGGGGAAAA
                           *****    * *************************************** *********

PacBio_read                ATAATAAGGTGAACGTATTGCATACCTATAGGCAAATGTGATTGTATTATCTGTAGAATG
SB_Mito_rep                ATAATAAGGTGAACGTATTGCATACCTATAGGCAAATGTGATTGTATTATCTGTAGAATG
                           ************************************************************

PacBio_read                GGTGTAGGCAAATATGATTATACTCCTTAAATTGGTTTAGTCTATAGGGGTTGTATTTAG
SB_Mito_rep                GGTGTAGGCAAATATGATTATACTCCTTAAATTGGTTTAGTCTATAGGGGTTGTATTTAG
                           ************************************************************

PacBio_read                AGTTCGTAGTTTAAGGTCTAAGGATTCTAAAGCAAATGTGATTGTACTCCTAAAATTATG
SB_Mito_rep                AGTTCGTAGTTTAAGGTCTAAGGATTCTAAAGCAAATGTGATTGTACTCCTAAAATTATG
                           ************************************************************
```

```
PacBio_read      TTAGTCTATAGAGGGTGTATTTA-GGTTTATGTTTAAGGTCTAAGGATTCTAAAGCAAAT
SB_Mito_rep      TTAGTCTATAGAGGGTGTATTTAGGGTTTATGTTTAAGGTCTAAGGATTCTAAAGCAAAT
                 *********************** ************************************

PacBio_read      GTGATTGTACTCCTAAAATTATGTTAGTCTATAGA-GGTGTATTTTAAAGATAAATGCAT
SB_Mito_rep      GTGATTGTACTCCTAAAATTATGTTAGTCTATAGAGGGTGTATTTTAAAGATAAATGCAT
                 *********************************** ************************

PacBio_read      TTTAATTTCTTGTAAGGGGGATGTACAACAAATTTGTTTAAATTTGGATTTTATGCTTTA
SB_Mito_rep      TTTAATTTCTTGTAAGGGGGATGTACAACAAATTTGTTTAAATTTGGATTTTATGCTTTA
                 ************************************************************

PacBio_read      ATATTGCTTTCAATTGAGGTAAATTTAATGATATTTTTATATTTTTATGTATTCAATTGT
SB_Mito_rep      ATATTGCTTTCAATTGAGGTAAATTTAATGATATTTTTTATATTTTATGTATTCAATTGT
                 **************************************    ******************

PacBio_read      GGTTATATTTTAAGGTATAAAAAGTTGTTGTTTCTGTAATCTTTTTGTTTTTTTTTTGGT
SB_Mito_rep      GGTTATATTTTAAGGTATAAAAAGTTGTTGTTTCTGTAATCTTTTTTGTTTTTTTTTGGT
                 **********************************************  ************

PacBio_read      GGTGGTAAGTTTAGTTGTATTTTAGTTCCAATAAAAATTTTTTGTAAAAAGTATGTTTTA
SB_Mito_rep      GGTGGTAAGTTTAGTTGTATTTTAGTTCCAATAAAAATTTTTTGTAAAAAGTATGTTTTA
                 ************************************************************

PacBio_read      ATTCATTTATATAT-ATATATTATAATATAATATAATGTAAAATAAGTATAGTATAGTAG
SB_Mito_rep      ATTCATTTATATATAATATATTATAATATAGTATAATGTAAAATAAGTATAGTATAGTAG
                 ************** *************** *****************************

PacBio_read      GGGGAAAAATAATAAGGTGAGCGTATCGCATACCTATAGGCAAATGTGATTGTATTATCT
SB_Mito_rep      GGGGAAAAATAATAAGGTGAGCGTATTGCATACCTATAGGCAAATGTGATTGTATTATCT
                 ************************** *********************************

PacBio_read      GTAGAATGGGTGTAGGCAAATATGATTATACTCCTTAAATTGGTTTAGTCTATAGGGGTT
SB_Mito_rep      GTAGAATGGGTGTAGGCAAATATGATTATACTCCTTAAATTGGTTTAGTCTATAGGGGTT
                 ************************************************************

PacBio_read      GTATTTAGAGTTCGTAGTTTAAGGTCTAAGGATTCTAAAGCAAATGTGATTGTACTCCTA
SB_Mito_rep      GTATTTAGAGTTCGTAGTTTAAGGTCTAAGGATTCTAAAGCAAATGTGATTGTACTCCTA
                 ************************************************************

PacBio_read      AAATTATGTTAGTCTATAGAGGGTG--TTTTAAAG--AAATGCATTTTAATTTCTTGTAA
SB_Mito_rep      AAATTATGTTAGTCTATAGAGGGTGTATTTTAAAGATAAATGCATTTTAATTTCTTGTAA
                 *************************  ********  ***********************

PacBio_read      GGGGGATGTACAACAAATTTGTTTAAATTTGGA-TTTATGCTTTAATATTGCTTTCAATT
SB_Mito_rep      GGGGGATGTACAACAAATTTGTTTAAATTTGGATTTTATGCTTTAATATTGCTTTCAATT
                 ********************************* **************************

PacBio_read      GA-GTAAATTTAATGAT-TTTTTTATATTTT-----------------------------
SB_Mito_rep      GAGGTAAATTTAATGATATTTTTTATATTTTTATGTATTCAATTGTGGTTATATTTTAAG
                 ** ************** *************

PacBio_read      ------------------------------------------------------------
SB_Mito_rep      GTATAAAAAGTTGTTGTTTCTGTAATCTTTTTTGTTTTTTTTGGTGGTGGTAAGTTTAGT

PacBio_read      ------------------------------------------------------------
SB_Mito_rep      TGTATTTTAGTTCCAATAAAAATTTTTTGTAAAAAGTATGTTTTAATTCATTTATATATA

PacBio_read      ------------------------------------------------------------
SB_Mito_rep      ATATATTATAATATAATATAATGTAAAATAAGTATAGTATAGTAGGGGGGAAAAATAATA

PacBio_read      ------------------------------------------------------------
SB_Mito_rep      AGGTGAGCGTATCGCATTCCTATAGGCAAATGTGATTGTATTATCTGTAGAATGGGTGTA

PacBio_read      ------------------------------------------------------------
SB_Mito_rep      GGCAAATATGATTATACTCCTTAAATTGGTTTAGTCTATAGGGTTGTATTTAGAGTTCGT

PacBio_read      ------------------------------------------------------------
SB_Mito_rep      AGTTTAAGGTCTAAGGATTCTAAAGCAAATGTGATTGTACTCCTAAAATTATGTTAGTCT
```

```
PacBio_read      ------------------------------------------------------------
SB_Mito_rep      ATAGAGGGTGTATTAGGGTTTGTGTTTGTTAGGAATGGTTAAATAGTTGGATAGAAAGGC

PacBio_read      ------------------------------------------------------------
SB_Mito_rep      TTATAGTAAGCTTCACCATCGAATGGTGAGTAAGATTTAAAAATCTCTATTTATATCTGT

PacBio_read      ------------------------------------------------------------
SB_Mito_rep      TTTGTTTTTGTTTTTTTTTTTTTGTGGTGGTAAGTTTAGTTGTATTTTAGTTCCAATAAA

PacBio_read      ------------------------------------------------------------
SB_Mito_rep      AATTTTTGTAAAAGTATGTTTTAATTCATTTATATATATATTATAAATAATATAATATAA

PacBio_read      ------------------------------------------------------------
SB_Mito_rep      TAAATAAGTATAGTATAGTAGGGGGGAAAAATAATAAGGTGAACGTATTGCATACCTATA

PacBio_read      ------------------------------------------------------------
SB_Mito_rep      GGCAAATGTGATTGTATTATCTGTAGAATGGGTGTAGGCAAATATGATTATACTCCTTAA

PacBio_read      ------------------------------------------------------------
SB_Mito_rep      ATTGGTTTAGTCTATAGGGTTGTATTTAGAGTTCGTAGTTTAAGGTCTAAGGATTCTAAA

PacBio_read      ------------------------------------------------------------
SB_Mito_rep      GCAAATGATTGTACTCCTAAAATTATGTTAGTCTATAGAGGGTGTATTTAGGGTTTATGT

PacBio_read      ------------------------------------------------------------
SB_Mito_rep      TTAAGGTCTAAGGATTCTAAAGCAAATGTGATTGTACTCCTAAAATTATGTTAGTCTATA

PacBio_read      --------------------------------------------ATGTA-----------
SB_Mito_rep      GAGGGTATTTTAAAGATAAATGCATTTTAATTCTTGTAAGGGGGATGTACAACAAATTTG
                                                             *****

PacBio_read      ------------------------------------------------------------
SB_Mito_rep      TTTAAATTTGGATTTTATGCTTTAATATTGCTTTCAATTGAGGTAAATTTAATGATATTT

PacBio_read      ----------------TCAATTGTGGTTATA--TTAAGGTAT-AAAAGTTGTTG--TCTG
SB_Mito_rep      TTTTATATTTTTGTATTCAATTGTGGTTATATTTTAAGGTATAAAAAGTTGTTGTTTCTG
                                 ***************  ********* ***********  ****

PacBio_read      TAATC----------------TTTTTGTTTTTTTTTTGGTGGTGGTAAGTTTAGTTGTAT
SB_Mito_rep      TAATCNNNNNNNNNNNNNNCTTTTTTGTTTTTTTTTTGGTGGTGGTAAGTTTAGTTGTAT
                 *****                ***************************************

PacBio_read      TTTAGTTCCAATAAAAATTTTTGTAAAAAGTATGTTTTAATTCATTTATATATAATATA
SB_Mito_rep      TTTAGTTCCAATAAAAATTTTTGTAAAAAGTATGTTTTAATTCATTTATATATAATATA
                 ************************************************************

PacBio_read      ATATAATAT------ATATAATG---AATAAGTATAGTATAGTAGGGGGAAAAATAATAA
SB_Mito_rep      TTATAATATAATATAATATAATGTAAAATAAGTATAGTATAGTAGGGGGAAAAATAATAA
                  ********      ********   **********************************

PacBio_read      GGTGAGCGTATCGCATACCTATAGGCAAATGTGATTGTATTATCTGTAGAATGGGTGTAG
SB_Mito_rep      GGTGAACGTATTGCATACCTATAGGCAAATGTGATTGTATTATCTGTAGAATGGGTGTAG
                 ***** ***** ************************************************

PacBio_read      GCAAATATGATTATACTCCTTAAATTGGTTTAGTCTATAGGGGTTGTATTTAGAGTTCGT
SB_Mito_rep      GCAAATATGATTATACTCCTTAAATTGGTTTAGTCTATAGGGGTTGTATTTAGAGTTCGT
                 ************************************************************

PacBio_read      A-----------------------------------------------------------
SB_Mito_rep      AGTTTAAGGTCTAAGGATTCTAAAGCAAATGTGATTGTACTCCTAAAATTATGTTAGTCT
                 *

PacBio_read      ------------------------GTTTAAGGTCTAAGGATTCTAAAGCAAATGTGATTG
SB_Mito_rep      ATAGAGGGTGTATTTAGGGTTTATGTTTAAGGTCTAAGGATTCTAAAGCAAATGTGATTG
                                         ************************************

PacBio_read      TACTCCTAAAATTATGTTAGTCTATAGAGGGTGTATTTTAAAGATAAATGCATTTTAATT
```

```
SB_Mito_rep     TACTCCTAAAATTATGTTAGTCTATAGAGGGTGTATTTTAAAGATAAATGCATTTTAATT
                ************************************************************

PacBio_read     TCTTGTAAGGGGGATGTACAACAAATTTGTTTAAATTTGGATTTTATGCTTTAATATTGC
SB_Mito_rep     TCTTGTAAGGGGGATGTACAACAAATTTGTTTAAATTTGGATTTTATGCTTTAATATTGC
                ************************************************************

PacBio_read     TTTCAATTGAGGTAAATTTAATGATA-TTTTTTATATTTTTATGTATTCAATTGTGGTTA
SB_Mito_rep     TTTCAATTGAGGTAAATTTAATGATATTTTTTTATATTTTTATGTATTCAATTGTGGTTA
                ************************** *********************************

PacBio_read     TATTTTAAGGTAT-----------------------------------------------
SB_Mito_rep     TATTTTAAGGTATAAAAAGTTGTTGTTTCTGTAATCTTTTTGTTTTTTTTTTGGTGGTG
                *************

PacBio_read     ------------------------------------------------------------
SB_Mito_rep     GTAAGTTTAGTTGTATTTTAGTTCCAATAAAAATTTTTTGTAAAAAGTATGTTTTAATTC

PacBio_read     ------------------------------------------------------------
SB_Mito_rep     ATTTATATAAATATATTATAATATAGTATAATGTAAAAATATAGTATAGTAGGGGGGAAA

PacBio_read     ------------------------------------------------------------
SB_Mito_rep     AATAATAAGGTGAGCGTATTGCATACCTATAGGCAAATGTGATTGTATTATCTGTAGAAT

PacBio_read     ------------------------------------------------------------
SB_Mito_rep     GGGTGTAGGCAAATATGATTATACTCCTTAAATTGGTTTAGTCTATAGGGGTTGTATTTA

PacBio_read     ------------------------------------------------------------
SB_Mito_rep     GAGTTCGTAGTTTAAGGTCTAAGGATTCTAAAGCAAATGTGATTGTACTCCTAAAATTAT

PacBio_read     ------------------------------------------------------------
SB_Mito_rep     GTTAGTCTATAGAGGGTGTATTTTAAAGATAAATGCATTTTAATTTCTTGTAAGGGGGAT

PacBio_read     ------------------------------------------------------------
SB_Mito_rep     GTACAACAAATTTGTTTAAATTTGGATTTTATGCTTTAATATTGCTTTCAATTGAGGTAA

PacBio_read     ----------------------------------------------------------AA
SB_Mito_rep     ATTTAATGATATTTTTTTATATTTTTATGTATTCAATTGTGGTTATATTTTAAGGTATAAA
                                                                          **

PacBio_read     AAGTTGTTGTTTCTGTAATTTTTTT-----------------------------------
SB_Mito_rep     AAGTTGTTGTTTCTGTAATCTTTTTTGTTTTTTTTTTGGTGGTGGTAAGTTTAGTTGTATT
                ******************* *****

PacBio_read     ------------------------------------------------------------
SB_Mito_rep     TTAGTTCCAATAAAAAATTTTTTGTAAAAAGTATGTTTTAATTCATTTATATATAATATAT

PacBio_read     ------------------------------------------------------------
SB_Mito_rep     TATAATATAATATATAATGTAAAATAAGTATAGTATAGTAGGGGGGAAAAATAATAAGGT

PacBio_read     ------------------------------------------------------------
SB_Mito_rep     GAGCGTATCGCATTCCTATAGGCAAATGTGATTGTATTATCTGTAGAATGGGTGTAGGCA

PacBio_read     ------------------------------------------------------------
SB_Mito_rep     AATATGATTATACTCCTTAAATTGGTTTAGTCTATAGGGGTTGTATTTAGAGTTCGTAGT

PacBio_read     ------------------------------------------------------------
SB_Mito_rep     TTAAGGTCTAAGGATTCTAAAGCAAATGTGATTGTACTCCTAAAATTATGTTAGTCTATA

PacBio_read     ------------------------------------------------------------
SB_Mito_rep     GAGGGTGTATTTAGGGTTTGTGTTTGTTAGGAATGGTTAAATAGTTGGATAGAAAGGCTT

PacBio_read     ------------------------------------------------------------
SB_Mito_rep     ATAGTAAGCTTCACCATCGAATGGTGAGTAAGATTTAAAAATCTCTATTTATAGTCTTGT
```

```
PacBio_read      -----------TTTTTTTTTGGTGGTGGTAAGTTTAGTTGTATTTTAGTTCCAATAAAAA
SB_Mito_rep      TTTTGTTTTTGTTTTTTTTTGGTGGTGGTAAGTTTAGTTGTATTTTAGTTCCAATAAAAA
                            *************************************************

PacBio_read      TTTTTTGTAAAAAGTATGTTTTAATTCATTTATATATAATATA-TATAATAT-ATATAAT
SB_Mito_rep      TTTTTTGTAAAAAGTATGTTTTAATTCATTTATATATAATATATTATAATATAATATAAT
                 ******************************************* ******** *******

PacBio_read      GTAAAATAAGTATAGTATAGTAGGGGGGAAAAATAATAAGGTGAGCGTATCGCATACCTA
SB_Mito_rep      GTAAAATAAGTATAGTATAGTAGGGGGGAAAAATAATAAGGTGAGCGTATCGCATACCTA
                 ************************************************************

PacBio_read      TAGGCAAATGTGATTGTATTATCTGTAGAATGGGTGTAGGCAAATATGATTATACTCCTT
SB_Mito_rep      TAGGCAAATGTGATTGTATTATCTGTAGAATGGGTGTAGGCAAATATGATTATACTCCTT
                 ************************************************************

PacBio_read      AAATTGGTTTAGTCTATAGGGGTTGTATTTAGAGTTCGTAGTTTAAGGTCTAAGGATTCT
SB_Mito_rep      AAATTGGTTTAGTCTATAGGGGTTGTATTTAGAGTTCGTAGTTTAAGGTCTAAGGATTCT
                 ************************************************************

PacBio_read      AAAGCAAATGTGATTGTACTCCTAAAATTATGTTAGTCTATAGAGGGTGTATTTTAAAGA
SB_Mito_rep      AAAGCAAATGTGATTGTACTCCTAAAATTATGTTAGTCTATAGAGGGTGTATTTTAAAGA
                 ************************************************************

PacBio_read      TAAATGCATTTTAATTTCTTGTAAGGGGGATGTACAACAAATTTGTTTAAATTTGGATTT
SB_Mito_rep      TAAATGCATTTTAATTTCTTGTAAGGGGGATGTACAACAAATTTGTTTAAATTTGGATTT
                 ************************************************************

PacBio_read      TATGCTTTAATATTGCTTTCAATTGAGGTAAATTTAATGATATTTTTTATATTTTTATGT
SB_Mito_rep      TATGCTTTAATATTGCTTTCAATTGAGGTAAATTTAATGATATTTTTTATATTTTTATGT
                 ************************************************************

PacBio_read      ATTCAATTGTGGTTATATTTTAAGGTATAAAAAGTTGTTGTTTCTGTAATCTTTTTTG-T
SB_Mito_rep      ATTCAATTGTGGTTATATTTTAAGGTATAAAAAGTTGTTGTTTCTGTAATCTTTTTTGTT
                 ********************************************************** *

PacBio_read      TTTTTTTTGGTGGTGGTAAGTTTAGTTGTATTTTAGTTCCAATAAAAATTTTTTGTAAAA
SB_Mito_rep      TTTTTTTTGGTGGTGGTAAGTTTAGTTGTATTTTAGTTCCAATAAAAATTTTTTGTAAAA
                 ************************************************************

PacBio_read      AGTATGTTTTAATTCATTTATATATAATATATTATAATAT--TATAATGTAAAATAAGTA
SB_Mito_rep      AGTATGTTTTAATTCATTTATATATAATATATTATAATATAGTATAATGTAAAATAAGTA
                 ****************************************  ******************

PacBio_read      TAGTATAGTA-GGGGGAAAAATAATAAGGTGAGCGTATCGCATACCTATAGGCAAATGTG
SB_Mito_rep      TAGTATAGTAGGGGGGAAAAATAATAAGGTGAGCGTATCGCATACCTATAGGCAAATGTG
                 ********** *************************************************

PacBio_read      ATTGTATTATCTGTAGAATGGGTGTAGGCAAATATGATTATACTCCTTAAATTGGTTTAG
SB_Mito_rep      ATTGTATTATCTGTAGAATGGGTGTAGGCAAATATGATTATACTCCTTAAATTGGTTTAG
                 ************************************************************

PacBio_read      TCTATAGGGGTTGTATTTAGAGTTCGTAGTTTAAGGTCTAAGGATTCTAAAGCAAATGTG
SB_Mito_rep      TCTATAGGGGTTGTATTTAGAGTTCGTAGTTTAAGGTCTAAGGATTCTAAAGCAAATGTG
                 ************************************************************

PacBio_read      ATTGTACTCCTAAAATTATGTTAGTCTATAGAGGGTGTATTTAGGGTTTATGTTTAAGGT
SB_Mito_rep      ATTGTACTCCTAAAATTATGTTAGTCTATAGAGGGTGTATTTAGGGTTTATGTTTAAGGT
                 ************************************************************

PacBio_read      CTAAGGATTCTAAAGCAAATGTGATTGTACTCCTAAAATTATGTTAGTCTATAGAGGGTG
SB_Mito_rep      CTAAGGATTCTAAAGCAAATGTGATTGTACTCCTAAAATTATGTTAGTCTATAGAGGGTG
                 ************************************************************

PacBio_read      TATTTAGGGTTTGTGTTTGTTAGGAATGGTTAAATAGTTGGATAGAAAGGCTTATAGTAA
SB_Mito_rep      TATTTAGGGTTTGTGTTTGTTAGGAATGGTTAAATAGTTGGATAGAAAGGCTTATAGTAA
                 ************************************************************

PacBio_read      GCTTCACCATCGAATGGTGAGTAAGATTTAAAAATCTCTATTTATAGAAGATATTGATAA
SB_Mito_rep      GCTTCACCATCGAATGGTGAGTAAGATTTAAAAATCTCTATTTATAGAAGATATTGATAA
                 ************************************************************

PacBio_read      TAAAACTAATGGATAAGTTT-TTATATATTTTTTAGGTTTATTATTTATCTTTTATGATT
SB_Mito_rep      TAAAACTAATGGATAAGTTTATTATATA-TTTTTAGGTTTATTATTTATCTTTTATGATT
                 ******************** ******* *******************************
```

```
PacBio_read     -TATTAGTACTCCTTTGTTAGTATTTATAGTATTTAAGTTTGTG-TTTTTTTAGTACTAA
SB_Mito_rep     ATATTAGTACTCCTTTGTTAGTATTTATAGTATTTAAGTTTGTGTTTTTTTTAGTACTAA
                 ******************************************* ***************

PacBio_read     GTTTGTTGAGTGGTGGGGTGTTTGTATATATTGTTATATGTTATTGTTTTTTATTGTTAA
SB_Mito_rep     GTTTGTTGAGTGGTGGGGTGTTTGTATATATTGTTATATGTTATTGTTTTTTATTGTTAA
                ************************************************************

PacBio_read     TAGTGATGTTTGTTTATTATTTGAATTTATAGGTTGATTATTCTAGTATATTATTATGAT
SB_Mito_rep     TAGTGATGTTTGTTTATTATTTGAATTTATAGGTTGATTATTCTAGTATATTATTATGAT
                ************************************************************

PacBio_read     TTTAG-TTAATTTATGTTA---TATATTCTGCGTAATAATTTGATGTATTAGTTTAGTTT
SB_Mito_rep     TTTAGTTTAATTTATGTTATGTTATATTCTGCGTAATAATTTGATGTATTAGTTTAGTTT
                ***** *************   **************************************

PacBio_read     ATGTTATGGTTATTTTGGTGGTTTAGATATTAAGTTAATTAAACTATGTGTTTCATGTGC
SB_Mito_rep     ATGTTATGGTTATTTTGGTGGTTTAGATATTAAGTTAATTAAACTATGTGTTTCATGTGC
                ************************************************************

PacBio_read     ACAAATTGCTGTATTTAAGTTACG--TATCTTTATTTATTATACTGGTTTAGGTCTGGTT
SB_Mito_rep     ACAAATTGCTGTATTTAAGTTACGCATATCTTTATTTATTATACTGGTTTAGGTCTGGTT
                ************************  **********************************

PacBio_read     ATCTAGTTTAGTAGGTTAGTTAGACTGCTTGCTTTCAAAGTAAGAGAGGATACTGTATTT
SB_Mito_rep     ATCTAGTTTAGTAGGTTAGTTAGACTGCTTGCTTTCAAAGTAAGAGGTGATACTGTATTT
                **********************************************  ************

PacBio_read     GGTCCTAGGCTGAGGTGTATGGAAGTAAAATTTGTTATCGCTGCTAACGATAATTTAGGA
SB_Mito_rep     GGTCCTAGGCTGAGGTGTATGGAAGTAAAATTTGTTATCGCTGCTAACGATAATTTAGGA
                ************************************************************

PacBio_read     -TATGTAGTGTAATT-TGCTTTCTTTATGTTTTATTAATTATATTGTTATAGCTAT-GAA
SB_Mito_rep     TTATGTAGTGTAATTCTGCTTTCTTTATGTTTTATTAATTATATTGTTATAGCTATGGAA
                 ************** **************************************** ***

PacBio_read     GAAACAAAGAAGAAGAAGAAGAGAGTTAAGAAGGAAGTTTCTA-AAAGAAGAAGA--ACT
SB_Mito_rep     GAAACAAAGAAGAAGAAGAAGAGAGTTAAGAAGGAAGTTTCTACAAAGAAGAAGAAGACT
                ******************************************* ***********  ***

PacBio_read     TCAGTTGGTGGAAGCGGTGGTTCTGGGTTTTGGAGGTAAGTTATTAGATTTATTAAATAA
SB_Mito_rep     TCAGTTGGTGGAAGCGGTGGTTCTGGGTTTT-GAGGTAAGTTATTAGATTTATTAAATAA
                ******************************* ****************************

PacBio_read     G--TTTAGGTGGATTTAAGAAGTCTATATGTTTAC-TATTAAGTTTGTAATTGGTAGTGT
SB_Mito_rep     GCTTTTAGGTGGATTTAAGAAGTCTATATGTTTACTTATTAAGTTTGTAATTGGTAGTGT
                *  ******************************** ************************

PacBio_read     AAGTGTGTTTCATTTTAATTTATATAAGTTTGTAGTATTGGGTTTAGATTCATTAAATAG
SB_Mito_rep     AAGTGTGTTTCATTTTAATTTATATAAGTTTGTAGTATTGGGTTTAGATTCATTAAATAG
                ************************************************************

PacBio_read     TTTAGTAGTTGATTTTTGTGTAAATGCTGTTGGTTATGGTTATTGGTTAATTTCTTTAGA
SB_Mito_rep     TTTAGTAGTTGATTTTTGTGTAAATGCTGTTGGTTATGGTTATTGGTTAATTTCTTTAGA
                ************************************************************

PacBio_read     TCATAAGCGGATAAGTTTAGTGTATATTATTTTAGGTTTATGAGGTGGTTTTATTGGTCT
SB_Mito_rep     TCATAAGCGGATAAGTTTAGTGTATATTATTTTAGGTTTATGAGGTGGTTTTATTGGTCT
                ************************************************************

PacBio_read     T-GTTTAAGGCTTCTAATTCGGTTAAATTTATGTGATCCATATTATAATTTGGTTTCGTT
SB_Mito_rep     TGGTTTAAGGCTTCTAATTCGGTTAAATTTATGTGATCCATATTATAATTTGGTTTCGTT
                * **********************************************************

PacBio_read     AGAGGTTTATAAATTTTTGATTACTAATCATGGTATAGCTATGA-TTTTTTTTTTTTGAT
SB_Mito_rep     AGAGGTTTATAAATTTTTGATTACTAATCATGGTATAGCTATGATTTTTTTTTTTTTGAT
                ******************************************** ***************

PacBio_read     GCCGGTATTAATAGGT-GATTTGGT-AATATTTTCTTCC-TTTTTTTTATATATAGATGA
SB_Mito_rep     GCCGGTATTAATAGGTGGATTTGGTAAATATTTTCTTCCTTTTTTTTTATATATAGATGA
                **************** ******** ************* ********************

PacBio_read     TTTATTGTTACCTCGGTTGAATTCTTTTAGTTTATGGTTGATGATTCCTTCATTTTTTTA
SB_Mito_rep     TTTATTGTTACCTCGGTTGAATTCTTTTAGTTTATGGTTGATGATTCCTTCATTTTTTTA
                ************************************************************
```

```
PacBio_read      TATGGAGCTAAGTTTATATTATGGTTGTGGGGTT-GTTGAACGTTGTATCCTCCATTATC
SB_Mito_rep      TATGGAGCTAAGTTTATATTATGGTTGTGGGGTTGGTTGAACGTTGTATCCTCCATTATC
                 ********************************** *************************

PacBio_read      TATATCTGAGAATTCTGGCTTGGGTGTAGATTATTTGATGTTTTCGTTGCATTTAGCGGG
SB_Mito_rep      TATATCTGAGAATTCTGGCTTGGGTGTAGATTATTTGATGTTTTCGTTGCATTTAGCGGG
                 ************************************************************

PacBio_read      TGTGTCTAGATTGGTTGGTTCAATTAATTTTATTTCTACAATTATGAGTTGTGTAGATTT
SB_Mito_rep      TGTGTCTAGATTGGTTGGTTCAATTAATTTTATTTCTACAATTATGAGTTGTGTAGATTT
                 ************************************************************

PacBio_read      TAAGACTTCGATAATAATCTGATCATATTTTATTTACTTCAATTTTATTGTTACTGTCAT
SB_Mito_rep      TAAGACTTCGATAATAATCTGATCATA-TTTATTTACTTCAATTTTATTGTTACTGTCAT
                 *************************** ********************************

PacBio_read      TACCCAGTTTTAGCGGCTGGTATAACTATGTTTATTATATTGATCGTAAATTTGGTACTG
SB_Mito_rep      TA-CCAGTTTTAGCGGCTGGTATAACTATG-TTATTAT-TTGATCGTAAATTTGGTACTG
                 ** *************************** ******* *********************

PacBio_read      -TTTTTTTGAGCCGATGGGTGGTGGTGATCCGCTGTTATTTCAGCATTTATTTTGGTTTT
SB_Mito_rep      CTTTTTTTGAGCCGATGGGTGGTGGTGATCCGCTGTTATTTCAGCATTTATTTTGGTTTT
                  ***********************************************************

PacBio_read      TTGGGCATCCTGAGGTGTATGTTTTGATTCTGCCAGGATTC-GTATAGTTAGTCATATAT
SB_Mito_rep      TTGGGCATCCTGAGGTGTATGTTTTGATTCTGCCAGGATTCGGTATAGTTAGTCATATAT
                 ***************************************** ******************

PacBio_read      GTATGAGAATTAGTAATAATGATTCATCATTTGGATATTATGGATTAATTTGTGCTATGG
SB_Mito_rep      GTATGAGAATTAGTAATAATGATTCATCATTTGGATATTATGGATTAATTTGTGCTATGG
                 ************************************************************

PacBio_read      CTTCAATAGTTTGTTTAGGTAGTGTAGTTT-GGCTCACCATATGTTTATGGTTGGTTTGG
SB_Mito_rep      CTTCAATAGTTTGTTTAGGTAGTGTAGTTTGGGCTCACCATATGTTTATGGTTGGTTTGG
                 ****************************** *****************************

PacBio_read      ATTA-TTGACAGCTATATTTTTTAGTTCTGTGACTCATGA-TATAGGGATTCCTAC-GGT
SB_Mito_rep      ATTATTTGACAGCTATATTTTTTAGTTCTGTGACT-ATGATTATAGGGATTCCTACGGGT
                 **** ****************************** **** *************** ***

PacBio_read      ATAAAGGTTTTTTCTTGATTATATATGCTTAAAAGATGTAGGTCTACTCGTTGCTTGTCT
SB_Mito_rep      ATAAAGGTTTTTTCTTGATTATATATGCTTAAAAGATGTGG---TACTCGT---TTGTCT
                 *************************************** *   *******   ******

PacBio_read      GATGACTCCTGTTGTTATTATGGTGAGTTGGT-GGTTATATACCTTTGTCATCTAACAAT
SB_Mito_rep      GA----TCCTG----TGTTATGGTG-GTTGGTGGGTTTTATATTTTTGT----TTACAAT
                 **    *****    * ******** ****** **** ****  *****    * *****

PacBio_read      CGTGGTAGGGTGGTGTACCTGGC-TAGCTTTTTTCTTGTTCTTTTCTACTATTGGACATT
SB_Mito_rep      -------AGGGGGTGTTACTGGTGTAGCTTTGTCGGCTTCCT-------CATTAGACATA
                         ** *****  ****  ******* *     * **        *** *****

PacBio_read      ATTATTCATGATATTTGTTTTGTTGTCATGTTCTGCCATTATGTTCTCTTATTCTTTAGA
SB_Mito_rep      TTATTTCATGATACTTGGTTTGTCGTTGCTCATTTTCATTATGTTCT----TTCGTTAGG
                  *  ********* *** ***** **       *  ***********    *** ****

PacBio_read      TATTGCTATTTAATAGATAGTCGGTCGACTAAATTGATAGATTGGTTTATTTGTATTTGT
SB_Mito_rep      TTCT-----------TATAGTAGGGTGATAATAATGTTGG-----------TATGATGGT
                 *  *            ***** **  **  * * ** * *           * *  * **

PacBio_read      GGTCCTTTTTATAAAATTTGATAGAGTACATGTAATGAATTATAGTTATCTCCTTACAGT
SB_Mito_rep      GGCCTTT----TATAATTGGATACAGT-------ATGA---ATAAGTATCTCCTACAGGG
                 ** * **    ** **** **** ***       ****   ***  ********    *

PacBio_read      CTATGATTTATTGTTCGATGGTT-GATTTTATATTTG-TATTTTCCTATGCATGAACTAT
SB_Mito_rep      TCATTGATTATTG-TCGATGGTTGGATTTAATCTTTGTTTTTTTCCTATGCAT----TAT
                   **   ****** ********* ***** ** **** * *************    ***

PacBio_read      TTATGGTTATTCGTATGATGGTTTGCCTCGTTCGGGTAGGTGTCTTATGTATGTGTTGTT
SB_Mito_rep      TTA--GGTATTC-----ATGGTTTGCCTCG-TCGGGTAAGT-TGCTATGATTGTGAGTAT
                 ***  * *****     ************* ******* ** *  ****  ****    *

PacBio_read      TAT--GGTTTATATACTGAGAAGATTATATTGTGTCGGTGTGTTATATTTCTGTTACT--
SB_Mito_rep      TATTGGGTTAAAGTACTGAGAAG--TATA----GGTGGTGTGTTA----TCTGTTACTAG
                 ***  **** *  **********  ****    *  *********    *********

PacBio_read      TGCTTTCGTAGTATGTTTTTAATTCTTTAGAGCAGAGTCATT------------------
```

```
SB_Mito_rep      TGCTTTCGTGT--TGTTTTTTATTCTTTG----AGAGTCATTGAGTGTCTGTAATCGTAT
                 *********    ******* *******     *********

PacBio_read      -------------------------TGTA-----------------------GTGTCATC
SB_Mito_rep      TATAGGATTATGGGGATCTGGCAGATGTATAACTAAAGTTATGGTTGTGCCGGTTCCAGC
                                          ****                       **  ** *

PacBio_read      TTAAATACGTTTATA-----------------------------------TATTA-----
SB_Mito_rep      TCATATA-ACTTATATTTGTGGTGGTAAGGTGTGATTTAATAATAAGTAGTATTATGGCA
                 * * ***   *****                                   *****

PacBio_read      ---------TTTTTTATGGGTAG----ATCGGCTGAATAGATATATTCCATT--------
SB_Mito_rep      TGCTTTATTTTTTTTATTTGTAGTAGTATAGGTATAGCAGGTGTAGTTTATTTATAAAAT
                          ********  ****    ** **   *  ** * ** *  ***

PacBio_read      ----------TTAACTAATGTTA----------TGGTGC---------------------
SB_Mito_rep      ATTAGTTTTGTAAACTAATGATAGTGTATGATGTGGCACTTATCTGTTTTCGTGTGTACC
                           * ******** **          ***  *

PacBio_read      -------------------------------TTGTGCCGTATAACTCAGAATC--TCAAT
SB_Mito_rep      AGATTGATTTAAAATTATTTTTAATGGTTTATTGTACCTTTTGTATCATGATCCTTTGAT
                                                ****  ** * *   ***  ***  *  **

PacBio_read      TATATTAACA----------------------------------TAGT------------
SB_Mito_rep      TGTAATTACAGTATTGTATGTCCCGAAAGGTTTACGATTTTATTTAGTCTAATCAAATAG
                 * ** * ***                                  ****

PacBio_read      ----------------------------------------------------ACTTAATA
SB_Mito_rep      CTGAATTTTTTGGCTAGAGGGGTTACAGGTTATTAAGTAGATTTCTAAAGAGACTAAATA
                                                                     *** ****

PacBio_read      TGTGGTTTATGTGTTACACGTGTC------------------------------------
SB_Mito_rep      AGTAG-TGATAAATTACCCGTGTAATCTTATATCTGGTTGGTAACTTGTTTATCGAGTTG
                  ** * * **   **** *****

PacBio_read      ---------TTGATCATATT----------------------------------------
SB_Mito_rep      TGTGGAAAGTTGATTGTGTTGATTTTCTAGAGCAGAGATTTAATTTTTGTTATGAGTAGT
                          *****  * **

PacBio_read      ----AATCAAAAT-----------------------------------------------
SB_Mito_rep      CTTAGATTAAGATTGTCTTTGGATTAGTAAGTTATTTACATAGCTTTGTTCACTACTGTG
                      ** ** **

PacBio_read      ---------------ACTCAGAACGTATCCTTACA-------------------------
SB_Mito_rep      GTAATATCTTGCTTGATTTGGTAGTTACCCTTATAATTGGTTGTATGATGATGAAGGAAT
                                * *  * *  ** ***** *

PacBio_read      ---------------TCAAAT---------------------------------------
SB_Mito_rep      TAGTAGGTTTATGGTTAAAATAGTTGTCTTTTTTTTCGGTGATGCTTTCGACTGTTTATT
                                * ****

PacBio_read      -----------------------------------------CCTC
SB_Mito_rep      AAAAACATTTCTATCTGATGAGGTTTAGATAGTATGACCTGCCCA
                                                          **
```

Coding region: Non-coding region: Assembly gap:

Figure J: Pairwise alignment of an error corrected PacBio read spanning the repetitive region of the *S. bovis* mitochondrial genome

The alignment shows a section of the *S. bovis* mitochondrial genome, centred on the mitochondrial region, aligned with a PacBio read found to span this region. The PacBio read was error corrected byCanu. The Mitochondrial sequence is rearranged such that it is centred on the repetitive region with 3kb of non-repetitive sequence included on either side. The PacBio read is the error corrected reverse complement of read m141015_175441_42138R_c100696632550000001823138502281592_s1_p0/116821/381_8437 RQ=0.777 id=41428_0

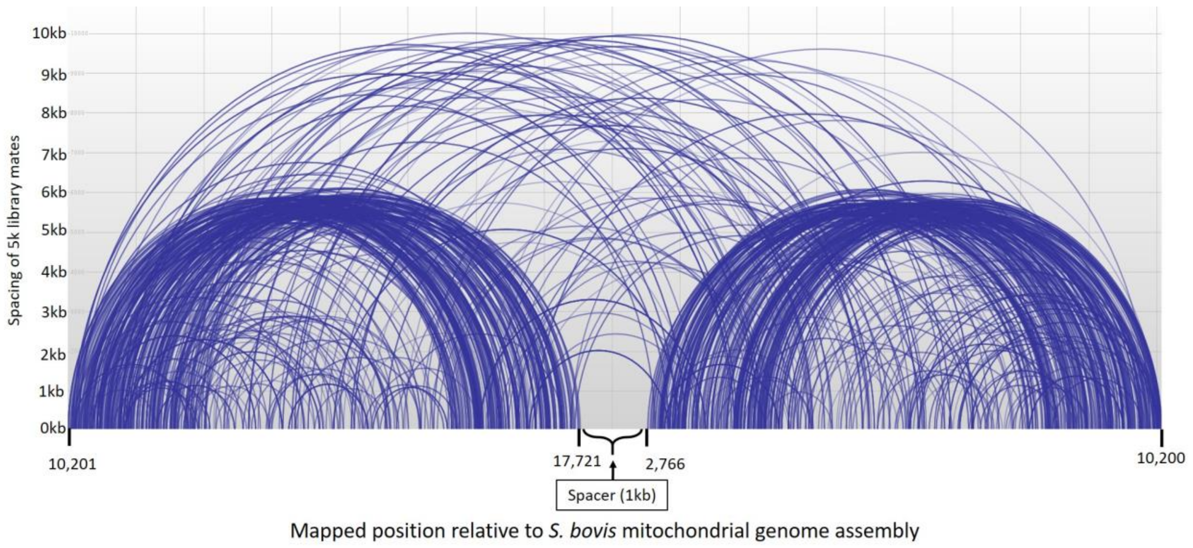

Figure K: 5kb mate pair library mapped to mitochondrial genome centered on the repetitive region

The figure shows the spacing of read-pairs mapped to the *S. bovis* mitochondrial genome in the reverse-forward orientation in the form of arcs with the height of the arcs representing the distance between the mapped reads in a pair (visualized in the Savant Genome Browser [4]). Read pairs spaced by more than 10kb or less than 1kb were omitted. The mitochondrial sequence was centered on the repetitive region with the region itself substituted for by a spacer comprised of 1,000 Ns.

Supplementary Tables

Table A: Overview of generated *S. bovis* sequence data.

Sequencing Platform	Average read length	Number of reads (M)	Bases (Gb)	GC content (%)
**Illumina**	90bp	623	56.1	35.91
**PacBio**	3.2kb	0.714	2.3	37.05

Table B: Summary of sequenced Illumina libraries.

Library	Insert size (bp)	Reads (M)	Sequenced bases (Gb)	High-Quality Reads (M)
Small insert / paired-end	200	158.8	14.3	137.0
Small insert / paired-end	500	174.4	15.7	133.5
Small insert / paired-end	800	91.6	8.2	58.2
Large insert / paired-end	2000	130.6	11.8	88.3
Large insert / paired-end	5000	68.0	6.1	45.4

Table C: Characterization of repetitive elements found in four *Schistosoma* genomes.

Repeat Type	*S. bovis*	*S. mansoni*	*S. haematobium*	*S. japonicum*
**Total interspersed repeats (%)**	35.83	39.66	34.44	36.85
**SINEs (%)**	2.57	3.25	2.19	2.26
**LINEs (%)**	17.38	19.4	19.14	18.69
**LTR elements (%)**	4.18	5.86	3.96	3.65
**DNA elements (%)**	0.56	0.76	0.56	0.94
**Unclassified (%)**	11.12	10.39	8.6	11.31

Table D: Characteristics of protein coding genes (CDSs) across four *Schistosoma* species.

Species	Total genes predicted	Average coding domain length (kb)	GC of coding sequences (%)	Average exon number per gene	Average exon length (bp)	Average intron length (kb)
S. bovis	11,631	1.2	36	4.8	256	1.95
S. haematobium	13,073	1.3	35	5.2	247	2.51
S. mansoni	11,774	1.4	36	6.4	225	2.43
S. japonicum	12,657	1.2	36	5.3	220	2.08

Table E: Conservation of parasite proteins previously linked to *S. haematobium*-induced cancer across *S. bovis* and *S. haematobium*. Proteins were mapped to the genome sequences using Exonerate.

Protein	Reference organism / Target organism[a]	Identity / Coverage of reference sequence	Description
**Estradiol 17beta-dehydrogenase**	*S. haematobium* / *S. bovis*	**98% / 100%**	Involved in the synthesis of estradiol. Proposed to be conducive of tumorigenesis in *S. haematobium,* potentially via estrogen receptor mediated cell proliferation leading to an increase of DNA mutations during replication
**Glycoprotein Omega-1**	*S. haematobium* / *S. bovis*	**92% / 100%**	Secretion from *Schistosoma* eggs thought to trigger Th2-type immune response leading to cancerous environment

[a]Query and target species of the Exonerate search.

Table F: Conservation of *Schistosoma* vaccine targets in *S. bovis*. Known vaccine targets were mapped to the *S. bovis* genome sequence using Exonerate.

Vaccine / Major antigen	Species targeted	Reference organism / Target organism[a]	Identity[b] / Coverage of reference sequence	Clinical development stage	Description
**Bilhvax / 28-kDa recombinant glutathione-S-transferase Sh28GST**	***S. haematobium***	*S. haematobium / S. bovis*	**97% / 100%**	Completed Phase 3 trial	Important for fatty acid metabolism and prostaglandin synthesis. Localised in tegument
**Sm14 / 14-kDa recombinant fatty acid binding protein Sm14**	***S. mansoni***	*S. mansoni / S. bovis*	**99% / 100%**	Successfully completed Phase 1 trial	Schistosomes are unable to synthesize fatty acids. Sm14 is required to import fatty acids from diet
**Sm-TSP-2 / 9-kDa recombinant tetraspanin Sm-TSP-2**	***S. mansoni***	*S. mansoni / S. bovis*	**34% / 89%**	Successfully completed Phase 1 trial	Expressed in tegument providing high levels of protection as recombinant vaccine in mouse models
**Sm-p80 (calpain)**	***S. mansoni* and *S. haematobium***	*S. mansoni* / *S. bovis*	**99% / 91%**	Pre-clinical development	Located in tegument and may play role in immune evasion by renewing surface membrane. Vaccines against large subunit Sm-p80 protected from *S. mansoni* and *S. haematobium* in model organisms

[a]Query and target species of the Exonerate search. [b]Sequence identity on amino acid level.

References

1. Doenhoff MJ, Cioli D, Utzinger J: **Praziquantel: mechanisms of action, resistance and new derivatives for schistosomiasis.** *Curr Opin Infect Dis* 2008, **21:**659-667.
2. Tebeje BM, Harvie M, You H, Loukas A, McManus DP: **Schistosomiasis vaccines: where do we stand?** *Parasit Vectors* 2016, **9:**528.
3. Merrifield M, Hotez PJ, Beaumier CM, Gillespie P, Strych U, Hayward T, Bottazzi ME: **Advancing a vaccine to prevent human schistosomiasis.** *Vaccine* 2016, **34:**2988-2991.
4. Fiume M, Williams V, Brook A, Brudno M: **Savant: genome browser for high-throughput sequencing data.** *Bioinformatics* 2010, **26:**1938-1944.
